# Immediate or delayed trial without catheter in acute urinary retention in males: A systematic review

**DOI:** 10.1002/bco2.369

**Published:** 2024-05-14

**Authors:** Veronika S. Christensen, Marius Skow, Signe A. Flottorp, Hilde Strømme, Ibrahimu Mdala, Odd Martin Vallersnes

**Affiliations:** ^1^ Faculty of Medicine University of Oslo Oslo Norway; ^2^ The Antibiotic Centre for Primary Care University of Oslo Oslo Norway; ^3^ Oslo Accident and Emergency Outpatient Clinic City of Oslo Health Agency Oslo Norway; ^4^ Department of General Practice University of Oslo Oslo Norway; ^5^ Division of Health Services Norwegian Institute of Public Health Oslo Norway; ^6^ Library of Medicine and Science University of Oslo Oslo Norway

**Keywords:** alpha‐blocker, benign prostatic hyperplasia, catheterization, trial without catheter, urinary retention

## Abstract

**Objective:**

To compare the success of establishing spontaneous micturition following immediate trial without catheter (TWOC) to delayed TWOC in males catheterized for acute urinary retention.

**Materials and methods:**

In this systematic review, we included studies reporting success rates of immediate TWOC or delayed TWOC (≤30 days) among males ≥18 years of age catheterized for acute urinary retention. We excluded studies on suprapubic catheterization, postoperative/perioperative catheterization and urinary retention related to trauma. We searched the following databases: MEDLINE, Embase, Cochrane Database of Systematic Reviews, Cochrane Central Register of Controlled Trials, Open Grey and Clinicaltrials.gov. The search was concluded on 30 November 2022. There were no restrictions on language or publication date. Risk of bias was assessed using the ROB 2.0 and ROBINS‐I tools. We did random‐effects restricted maximum likelihood model meta‐analyses. Certainty of evidence was assessed using GRADE.

**Results:**

We included 61 studies. In two randomized controlled trials (RCTs), both with some concerns for risk of bias, including in total 174 participants, the relative success rate was 1.22 (95% CI 0.84–1.76) favouring delayed TWOC. In two comparative cohort studies, both with serious risk of bias, including 642 participants, the relative success rate was 1.18 (0.94–1.47) favouring delayed TWOC. One study was excluded from this meta‐analysis because of critically low quality. Four studies reporting success rates for cohorts with immediate TWOC, all with serious risk of bias, including 409 participants, had an overall success rate of 47% (29–66). Fifty‐two studies reporting success rates for cohorts with delayed TWOC, all with serious risk of bias, including 12 489 participants, had an overall success rate of 53% (49–56). The certainty of the evidence was considered low for the RCTs and very low for the rest.

**Conclusion:**

There was a limited number of appropriately designed studies addressing the research question directly. The evidence favours neither approach.

## 1 INTRODUCTION

Acute urinary retention is a common urological emergency, characterized by the inability to pass urine, usually accompanied by escalating pain and distress.^1^, ^2^, ^3^, ^4^ The diagnosis is usually clinically obvious, with a typical history and an enlarged bladder on palpation or percussion. An ultrasound scan may verify the diagnosis. The patients are usually initially managed in primary care emergency settings or in hospital emergency departments (ED) by urethral catheterization.^2^, ^5^, ^6^, ^7^

Benign prostatic hyperplasia obstructing the urethra is the most common cause of acute urinary retention.^8^ Other causes are prostatic cancer, urethral stricture, blood clots, constipation, acute prostatitis, urethritis, anticholinergic and sympathomimetic drug effects, neurogenic causes and overfilling the bladder, often while under the influence of alcohol.^2^, ^8^, ^9^

After initial treatment with urethral catheterization, it is usually recommended to leave the catheter in place,^2^, ^5^ as acute urinary retention has been reported to occur in 50% of patients within a week if the bladder is merely emptied and the catheter immediately withdrawn.^10^ However, the longer the catheter remains in place, the higher the risk of complications such as infection, trauma, strictures and erosion.^11^, ^12^ Hence, trial without catheter (TWOC) is recommended after 2–7 days, though the optimal time has not been established.^2^, ^5^ When benign prostatic hyperplasia is the likely cause, initiating alpha‐blocker therapy before TWOC is beneficial and reduces the risk of recurrent acute urinary retention.^13^ Still, TWOC failure is reported among 40% of patients treated with an alpha‐blocker.^13^ Additionally, many men find the catheter cumbersome and inconvenient.^14^, ^15^ We wanted to investigate whether immediate TWOC could serve as an adequate treatment strategy.

### 1.1 Objectives

In this systematic review, we aim to compare the success rate of establishing spontaneous micturition following immediate TWOC to delayed TWOC, among adult males catheterized for acute urinary retention. We also compare the rate of complications and adverse events associated with both strategies, as well as patient and health care provider satisfaction and acceptability.

## 2 MATERIALS AND METHODS

We report this systematic review according to the PRISMA 2020 statement.^16^, ^17^

### 2.1 Eligibility criteria

We included any study, regardless of design, reporting success rates of establishing spontaneous micturition following immediate TWOC or delayed TWOC (≤30 days) among males ≥18 years of age catheterized for acute urinary retention. Studies reported in congress abstracts were also eligible for inclusion. We excluded editorials, guidelines, review papers, letters to the editor and case reports. Furthermore, we excluded studies on suprapubic catheterization, postoperative/perioperative urinary retention or catheterization, urinary retention related to trauma and placement of permanent indwelling catheter.

### 2.2 Information sources

On 11 May 2021, HS searched the databases MEDLINE (Ovid), Embase (Ovid), Cochrane Database of Systematic Reviews (Cochrane Library, Wiley), Cochrane Central Register of Controlled Trials (Cochrane Library, Wiley), OpenGrey and ClinicalTrials.gov. We subsequently searched the reference lists of the included reports after full‐text review for additional eligible studies. Abstract proceedings from urological meetings were covered through the database search to the extent that abstracts were indexed in the databases. On 30 November 2022, HS performed a new database search, updating and replacing the previous search. We then searched the reference lists of the newly included reports.

### 2.3 Search strategy

The search strategy was developed after a preliminary search and is described in Table S1. There were no restrictions on language or publication date. We validated the search strategy using five clearly eligible studies identified in the preliminary search. They were all found in the database search proper (Gas 2019, Bansal 2017, Fitzpatrick 2012, Klarskov 1987, Breum 1982^10^, ^12^, ^18^, ^19^, ^20^).

### 2.4 Study selection process

We used the Covidence tool for the selection process. Two researchers independently screened each title and abstract of the retrieved records. In case of disagreement, a third researcher was consulted to decide which reports to review in full text. VSC, MS and OMV participated in the screening. Next, two researchers independently reviewed each full‐text report for inclusion. In case of disagreement, they reached a consensus through discussion. VSC, MS and OMV participated in this process. When translation into English was required, we used Google Translate/Google Lens (Chinese, French, German, Korean, Russian and Spanish).

### 2.5 Data collection process

The Covidence tool was also used for the data collection. Two researchers independently collected data from each included study. Discrepancies were resolved through discussion. VSC, MS and OMV participated in the data collection. When translation into English was required, we used Google Translate/Google Lens (Chinese, Korean, Russian and Spanish). If studies included both male and female patients, we only collected data for males.

### 2.6 Data items

The primary outcome was successful TWOC. The secondary outcomes were complications, other adverse events, and patient and health care provider satisfaction and acceptability. We did not register adverse effects related to medication given as part of an intervention.

We also collected data on author, year of publication, country, setting, funding, design, inclusion/exclusion criteria, number of participants, patient characteristics [age, prostate volume, intravesical prostatic protrusion (IPP), detrusor wall thickness (DTW), lower urinary tract symptoms (LUTS), international prostate symptom score (IPSS), prostate‐specific antigen (PSA) serum level, C‐reactive protein (CRP) serum level, urinary tract infection (UTI), use of alpha‐blocker, type of alpha‐blocker used, residual volume voided], intervention details and definition of successful TWOC.

### 2.7 Risk of bias assessment

Two researchers independently assessed risk of bias for each study using the RoB 2.0 tool^21^ for randomized controlled trials (RCTs) and the ROBINS‐I tool^22^ for the rest of the studies. MS, SAF and OMV participated in this assessment. Disagreements were resolved through discussion.

The five domains assessed in the RoB 2.0 tool were the randomization process, deviations from intended interventions, missing outcome data, measurement of the outcome and selection of the reported results.^21^ In accordance with the RoB 2.0 tool rules, the overall risk of bias for a study was set at the level of the domain with the highest risk.

As requested in the ROBINS‐I tool, we specified a target randomized trial as ideal for our study: an individually randomized trial of males 18 years of age or older treated with urethral catheter for acute urinary retention, comparing immediate TWOC with delayed TWOC (defined as 1–30 days). The seven domains assessed in the ROBINS‐I tool were bias due to confounding, bias in selection of participants into the study, bias in classification of interventions, bias due to deviations from intended interventions, bias due to missing data, bias in measurement of outcomes and bias in selection of reported results.^22^ In accordance with the ROBINS‐I tool rules, the overall risk of bias for a study was set at the level of the domain with the highest risk.

### 2.8 Effect measures

For the primary outcome, the effect measure was the proportion of patients with successful TWOC. Complications and adverse events were presented as proportions. Patient satisfaction was measured using the International Prostate Symptom Score–Quality of Life Index (IPSS QoL). The IPSS QoL is a single question: ‘If you were to spend the rest of your life with your urinary condition just the way it is now, how would you feel about that?’, answered on an ordinal scale of 0–6, where 0 is delighted and 6 terrible.

### 2.9 Synthesis methods

We categorized the included studies according to design regarding the primary outcome: randomized and non‐randomized studies comparing immediate with delayed TWOC, and non‐comparative studies reporting only the success rate of immediate or delayed TWOC. Meta‐analyses were done separately for each category and presented in forest plots.

For the comparative studies, we calculated the relative risk of successful TWOC. If a study included several cohorts with different timing for delayed TWOC, these were combined into a joint cohort of delayed TWOC.

For the non‐comparative studies, we plotted cohorts with different co‐interventions as separate cohorts. We subcategorized cohorts according to whether alpha‐blockers were given or not. The success rate for each cohort was presented in forest plots for each subcategory.

The secondary outcomes were presented in a table including only the studies reporting on these outcomes. We did not perform any meta‐analysis for the secondary outcomes.

For the meta‐analyses, we used a random‐effects restricted maximum likelihood model in Stata. We quantified statistical heterogeneity by measuring the degree of inconsistency (*I* ^2^).

### 2.10 Reporting bias assessment

We assessed bias due to missing results in the included studies using the ROB 2.0 tool^21^ for RCTs and the ROBINS‐I^22^ tool for other studies.

### 2.11 Certainty assessment

We assessed the certainty of the evidence using GRADE,^23^ and we only formally assessed the comparative studies. The factors considered were design, risk of bias, inconsistency, indirectness and imprecision. Two researchers (SAF and OMV) independently assessed the certainty. We resolved any disagreements through discussion.

## 3 RESULTS

### 3.1 Study selection

**FIGURE 1:**
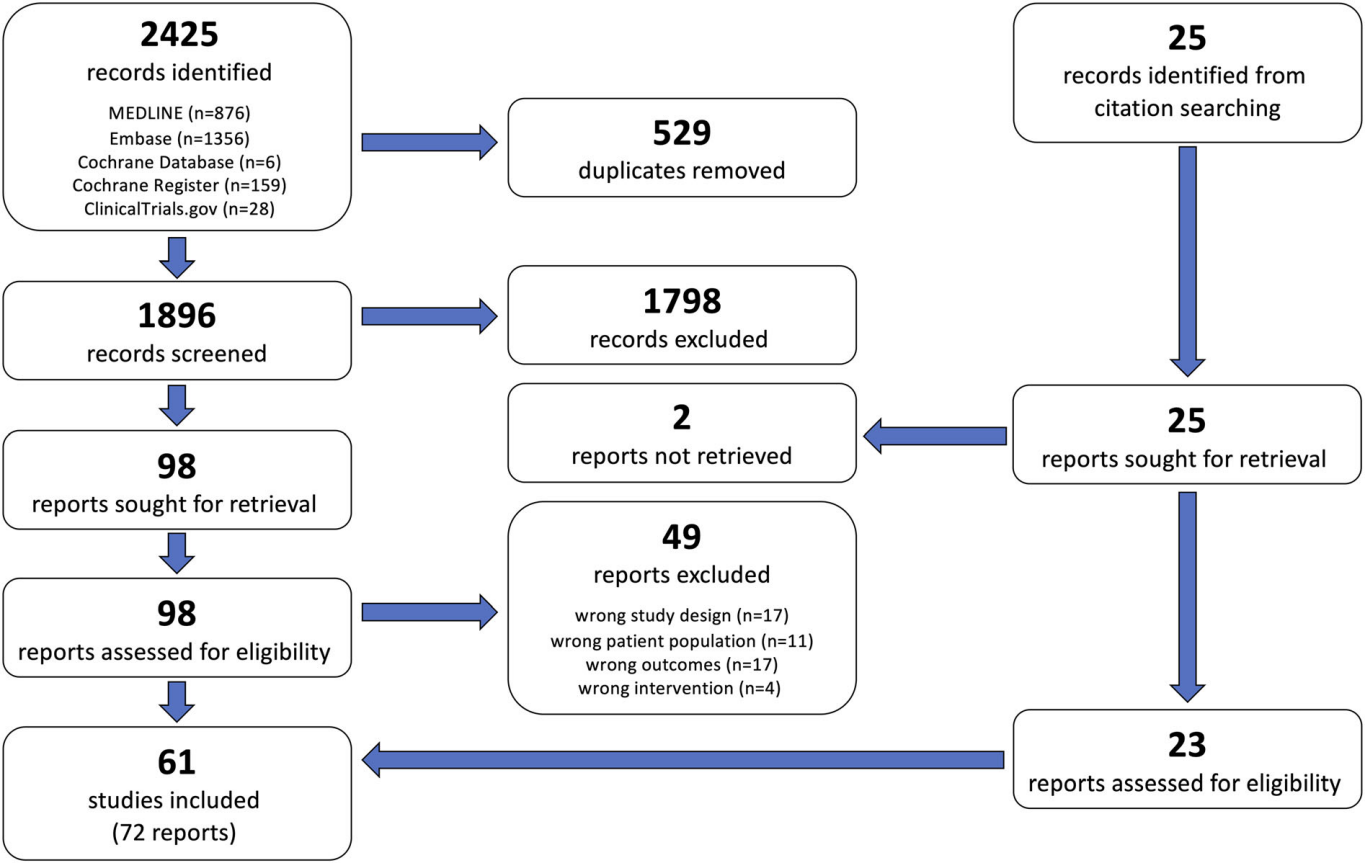
Flow diagram for the search and selection of studies, PRISMA 2020.^17^

We found 2425 records in the database search (PRISMA flow chart; Figure 1). After removing duplicates and screening, 49 reports were included. Another 25 reports were identified from the reference lists of the included studies. Two of these we were not able to retrieve in either abstract or full text (Pushkar 2004 and Unkert 2002^24^, ^25^), the remaining 23 were included. In total, we included 61 studies, reported in 72 articles/abstracts.

### 3.2 Study characteristics

We identified five comparative studies providing data for our primary outcome; two RCTs (Djavan 1998 and Taube 1989^26^, ^27^), one prospective comparative cohort study (Bouras 2018^28^) and two retrospective comparative cohort studies (Ko 2012 and Kim 2008^29^, ^30^). Alpha‐blockers were given in all three comparative cohort studies but not in the RCTs.^26^, ^27^, ^28^, ^29^, ^30^

Four studies reported only on immediate TWOC cohorts; one RCT comparing alpha‐blockers and placebo (Chan 1996^31^), two prospective cohort studies (Li 2009 and Klarskov 1987^20^, ^32^) and one retrospective cohort study (Breum 1982^10^). Alpha‐blockers were not given in Klarskov 1987 or Breum 1982, but in Li 2009, 60.1% of the participants were already on alpha‐blockers and an additional 16.8% were started on one.^10^, ^20^, ^32^

Finally, there were 52 studies reporting only cohorts with delayed TWOC. There were 18 RCTs comparing alpha‐blockers to placebo or comparing different regimens of alpha‐blockers and TWOC timing with or without other co‐interventions (Salem Mohamed 2018, Kara 2014, Maldonado‐Ávila 2014, Sharifi 2014, Zhengyong 2014, Elbendary 2013, Kumar 2013, Agrawal 2009, Tiong 2009, Al‐Hashimi 2007, Lucas 2005, McNeill 2004, Lorente Garín 2004, Hua 2003, Shah 2002, Bowden 2001, Perepanova 2001 and McNeill 1999^33^, ^34^, ^35^, ^36^, ^37^, ^38^, ^39^, ^40^, ^41^, ^42^, ^43^, ^44^, ^45^, ^46^, ^47^, ^48^, ^49^, ^50^, ^51^, ^52^, ^53^, ^54^, ^55^, ^56^, ^57^, ^58^) and one comparing different TWOC timing in the absence of alpha‐blockers (Lim 1999^59^). There were 19 prospective cohort studies of different regimens of alpha‐blockers and TWOC timing with or without other co‐interventions (Khadka 2021, Phuong Hoai 2021, Jha 2020, Vella 2019, Das 2018, Bansal 2017, Farelo‐Trejos 2017, Hagiwara 2016, Green 2014, Lodh 2013, Mahadik 2013, Fitzpatrick 2012, Bhomi 2011, Daly 2009, Pandit 2008, Mariappan 2007, Gopi 2006, Abeygunasekera 2001 and Kim 2001^12^, ^19^, ^60^, ^61^, ^62^, ^63^, ^64^, ^65^, ^66^, ^67^, ^68^, ^69^, ^70^, ^71^, ^72^, ^73^, ^74^, ^75^, ^76^, ^77^, ^78^), eight prospective cohort studies without alpha‐blockers (Kurniasari 2019, Ferdian 2016, Sharis 2013, Zeif 2010, Panda 2008, Tan 2003, Kumar 2000 and Hastie 1990^79^, ^80^, ^81^, ^82^, ^83^, ^84^, ^85^, ^86^, ^87^), four retrospective cohort studies with different regimens of alpha‐blockers (Gas 2019, Rasner 2009, Tsui 2008 and Park 2006^18^, ^88^, ^89^, ^90^), one retrospective cohort study without alpha‐blockers (Park 2012^91^) and one case–control study on alpha‐blockers (Tang 2015^92^).

The included studies are described in Table 1 and Tables S2 and S3.

**TABLE 1:** Study characteristics

Study	Design	Country	Participants	Inclusion	Interventions	TWOC success rate		
						*n*	*N*	%
RCTs comparing immediate TWOC with delayed TWOC								
Djavan 1998^26^	RCT, reported only in congress abstract	Austria	114	AUR	Immediate TWOC TWOC day 2 TWOC day 7	17 20 23	38 39 37	44.7 51.3 62.2 ns^a^
Taube 1989^27^	RCT	UK	60	AUR	Immediate TWOC TWOC 24 h TWOC 48 h	5 4 8	18 20 22	27.8 20.0 36.4 ns
Other studies comparing immediate TWOC with delayed TWOC								
Bouras 2018^28^	Prospective cohort study, reported only in congress abstract	Algeria	77	AUR	Immediate TWOC TWOC day 2 TWOC day 3 TWOC day 10	3 9 7 2	4 14 10 9	75.0 64.3 70.0 22.2 ns
Ko 2012^29^	Retrospective and prospective cohort study	Korea	515 28	AUR due to BPH, RV ≤ 1500 mL	Immediate TWOC + tamsulosin 0.2 mg TWOC day 7 + tamsulosin 0.2 mg Immediate TWOC + tamsulosin 0.2 mg TWOC day 7 + tamsulosin 0.2 mg	49 97 5 16	195 320 7 21	25.1 30.3 ns 71.4 76.2
Kim 2008^30^	Retrospective cohort study [Korean]	Korea	127	AUR	Immediate TWOC + tamsulosin 0.2 mg TWOC day 7 + tamsulosin 0.2 mg	30 36	62 65	48.4 55.4 ns^a^
Studies reporting success rate of immediate TWOC								
Li 2009^32^	Prospective cohort study	Hong Kong	143	AUR	Immediate TWOC (+ alpha‐blocker in 76.9%)	72	143	50.3
Chan 1996^31^	RCT, reported only in congress abstract	Hong Kong	29	AUR	Immediate TWOC + terazosin 10 mg Immediate TWOC + terazosin 5 mg Immediate TWOC + placebo	7 6 2	8 8 13	87.5 75.0 15.4
Klarskov 1987^20^	Prospective cohort study	Denmark	228	AUR	Immediate TWOC	76	173	43.9
Breum 1982^10^	Retrospective cohort study	Denmark	70	AUR due to BPH	Immediate TWOC	19	64	29.7
Studies reporting success rate of delayed TWOC								
Khadka 2021^60^	Prospective cohort study	Nepal	60	AUR due to BPH	TWOC day 7 + alfuzosin 10 mg	35	60	58.3
Phuong Hoai 2021^61^	Prospective cohort study	Vietnam	73	AUR due to BPH	TWOC day 3 + alfuzosin 10 mg	47	73	64.4
Jha 2020^62^	Prospective cohort study	India	90	AUR due to BPH, RV ≤ 1000 mL	TWOC day 3 + tamsulosin 0.4 mg	58	90	64.4
Gas 2019^18^	Retrospective cohort study	France	248	AUR	TWOC day 18 + alpha‐blocker	103	222	46.4
Kurniasari 2019^79^	Prospective cohort study	Indonesia	24	AUR due to BPH	TWOC day 5	13	24	54.2
Vella 2019^63^	Prospective cohort study	Italy	37	AUR	TWOC day 14 + alpha‐blocker + fluoroquinolone + Serenoa repens extract	11	37	29.7
Das 2018^64^	Prospective cohort study	India	90	AUR due to BPH	TWOC day 4 + tamsulosin 0.4 mg	51	90	56.7
Salem Mohamed 2018^33^	RCT	Egypt	60	AUR due to BPH, RV ≤ 1000 mL	TWOC day 3 + tamsulosin 0.4 mg + levofloxacin 500 mg TWOC day 7 + tamsulosin 0.4 mg + levofloxacin 500 mg	18 21	30 30	60.0 70.0
Bansal 2017^19^	Prospective cohort study	India	2188	AUR due to BPH	TWOC day 4 + alpha‐blocker	737	2188	33.7
Farelo‐Trejos 2017^65^	Prospective cohort study [Spanish]	Argentina	65	AUR due to BPH	TWOC day 10 + tamsulosin 0.4 mg	25	65	38.5
Ferdian 2016^80^ Jouwena 2016^81^	Prospective cohort study, reported only in congress abstracts	Indonesia	60	AUR due to BPH	TWOC ‘after a certain period’	26	60	43.3
Hagiwara 2016^66^	Prospective cohort study	Japan	80	AUR	TWOC day 14 + silodosin 4 mg ×2 + dutasteride 0.5 mg	47	80	58.8
Tang 2015^92^	Case–control study	Hong Kong	116	AUR due to BPH	TWOC day 4 + alfuzosin 10 mg TWOC day 4 + terazosin 2–4 mg	32 42	57 59	57.1 71.2
Green 2014^67^	Prospective cohort study	UK	187	AUR	TWOC day 13 + alpha‐blocker	63	123	51.2
Kara 2014^34^	RCT	Turkey	70	AUR due to BPH, RV 500–1500 mL	TWOC day 3 + tamsulosin 0.4 mg TWOC day 3 + tamsulosin 0.4 mg + alfuzosin 10 mg	19 27	35 35	54.3 77.1
Maldonado‐Ávila 2014^35^ Maldonado‐Ávila 2012^36^	RCT	Mexico	90	AUR due to BPH	TWOC day 5 + tamsulosin 0.4 mg TWOC day 5 + alfuzosin 10 mg TWOC day 5 + placebo	16 12 5	37 34 19	43.2 35.2 26.3
Sharifi 2014^37^	RCT	Iran	101	AUR due to BPH, RV ≤ 1000 mL	TWOC day 3 + tamsulosin 0.4 mg + sildenafil 50 mg TWOC day 3 + tamsulosin 0.4 mg + placebo	35 32	50 51	70.0 62.7
Zhengyong 2014^38^	RCT	China	845	AUR due to BPH	TWOC day 7 + tamsulosin 0.2 mg + finasteride 5 mg + bladder training TWOC day 7 + tamsulosin 0.2 mg + finasteride 5 mg + free drainage	287 278	440 405	62.5 68.6
Elbendary 2013^39^	RCT	Egypt	106	AUR due to BPH	TWOC day 7 + tamsulosin 0.4 mg + ketoconazole 200 mg TWOC day 7 + tamsulosin 0.4 mg + placebo	41 28	53 53	77.4 58.8
Kumar 2013^40^	RCT	India	60	AUR, RV 400–1000 mL	TWOC day 3 + silodosin 8 mg TWOC day 3 + placebo	23 11	30 30	76.7 36.7
Lodh 2013^68^	Prospective cohort study	India	83	AUR due to BPH	TWOC day 8 + tamsulosin 0.4 mg	35	83	42.2
Mahadik 2013^69^	Prospective cohort study	India	58	AUR due to BPH, RV < 1200 mL	TWOC day 2 + tamsulosin 0.4 mg	30	58	51.7
Sharis 2013^82^	Prospective cohort study	Malaysia	32	AUR	TWOC day 10	16	32	50.0
Fitzpatrick 2012^12^ Emberton 2008^70^ Desgrandchamps 2006^71^	Prospective cohort study	France, Korea, Pakistan, Philippines, Taiwan, Thailand, Vietnam, Colombia, Mexico, Venezuela, Algeria, Bahrain, Qatar, Kuwait, United Arab Emirates, Ireland	6074	AUR due to BPH	TWOC day 5 + alpha‐blocker	2866	4667	61.4
Park 2012^91^	Retrospective cohort study	Korea	299	AUR	TWOC day 9	216	269	80.3
Bhomi 2011^72^	Prospective cohort study	Nepal	64	AUR due to BPH	TWOC day 3 + tamsulosin 0.4 mg	28	64	43.8
Zeif 2010^83^	Prospective cohort study	UK	100	AUR	TWOC day 1	48	100	48.0
Agrawal 2009^41^	RCT	India	150	AUR due to BPH, RV 500–1500 mL	TWOC day 3 + alfuzosin 10 mg TWOC day 3 + tamsulosin 0.4 mg TWOC day 3 + placebo	33 35 18	50 50 50	66.0 70.0 36.0
Daly 2009^73^	Prospective cohort study	Ireland	72	AUR	TWOC day 28 + alpha‐blocker	27	72	37.5
Rasner 2009^88^	Retrospective cohort study [Russian]	Russia	232	AUR due to BPH	TWOC day 4 + alfuzosin 10 mg + tamsulosin 0.4 mg TWOC day 4 + alfuzosin 10 mg	87 41	136 96	64.0 42.7
Tiong 2009^42^	RCT	Singapore	64	AUR due to BPH, RV 500–1000 mL	TWOC day 2 + alfuzosin 10 mg TWOC day 2 + placebo	21 11	33 31	63.6 35.5
Panda 2008^84^	Prospective cohort study, reported only in congress abstract	India	36	AUR due to BPH	TWOC day 3	11	36	30.5
Pandit 2008^74^	Prospective cohort study	Nepal	45	AUR due to BPH	TWOC day 9 + alpha‐blocker	31	45	68.9
Tsui 2008^89^	Retrospective cohort study	Hong Kong	68	AUR due to BPH	TWOC day 3 + terazosin 2 mg TWOC day 3 + terazosin 4 mg	8 27	18 45	44.4 60.0
Al‐Hashimi 2007^43^	RCT	Iraq	245	AUR due to BPH RV 400–1500 mL	TWOC day 3 + alfuzosin 10 mg TWOC day 3 + placebo	71 36	114 110	62.3 32.7
Mariappan 2007^75^	Prospective cohort study	UK	57	AUR due to BPH, RV < 1500 mL	TWOC day 14 + alfuzosin 10 mg	25	57	43.9
Gopi 2006^76^	Prospective cohort study	UK	31	AUR	TWOC day 6 + alfuzosin 10 mg	19	31	61.3
Park 2006^90^	Retrospective cohort study [Korean]	Korea	455	AUR due to BPH	TWOC day 7 + alpha‐blocker	292	455	64.2
Lucas 2005^44^ Lucas 2002^45^	RCT	UK & Ireland	149	AUR due to BPH, RV 500–1500 mL	TWOC day 5 + tamsulosin 0.4 mg TWOC day 5 + placebo	24 17	71 70	33.8 24.3
Lorente Garín 2004^50^	RCT [Spanish]	Spain	40	AUR due to BPH	TWOC day 7 + doxazosin 4 mg TWOC day 7	12 5	20 20	60.0 25.0
McNeill 2004^46^ McNeill 2005^47^ Hargreave 2003^48^ McNeill 2003^49^	RCT	Belgium, Hungary, France, Ukraine, Netherlands, Poland, Russia, Bulgaria, UK, South Africa	360	AUR due to BPH, RV 500–1500 mL	TWOC day 2 + alfuzosin 10 mg TWOC day 2 + placebo	146 58	236 121	61.9 47.9
Hua 2003^51^	RCT [Chinese]	China	72	AUR due to BPH	TWOC day 3 + tamsulosin 0.4 mg TWOC day 3	22 10	36 36	61.1 27.8
Tan 2003^85^	Prospective cohort study	Singapore	100	AUR	TWOC day 2	46	100	46.0
Shah 2002^52^	RCT	UK	62	AUR	TWOC day 2 + alfuzosin 5 mg ×2 TWOC day 2 + placebo ×2	17 16	34 28	50.0 57.1
Abeygunasekera 2001^77^	Prospective cohort study	Sri Lanka	94	AUR due to BPH	TWOC day 7 + prazosin	56	94	59.6
Bowden 2001^53^	RCT, reported only in congress abstract	UK	49	AUR due to BPH	TWOC day 2 + tamsulosin 0.4 mg TWOC day 2 + placebo	19 7	30 19	63.3 36.8
Kim 2001^78^	Prospective cohort study	USA	33	AUR	TWOC day 7 + tamsulosin 0.4 mg	26	33	78.8
Perepanova 2001^54^	RCT [Russian]	Russia	36	AUR due to BPH	TWOC day 1 + doxazosin 4 mg TWOC day 1 + placebo	19 1	30 6	63.3 16.7
Kumar 2000^86^	Prospective cohort study	UK	40	AUR due to BPH	TWOC day 2	22	40	55.0
Lim 1999^59^	RCT	Singapore	79	AUR	TWOC day 1 TWOC day 2	18 28	35 44	51.4 63.6
McNeill 1999^55^ McNeill 2004^56^ McNeill 1998^57^ McNeill 2000^58^	RCT	UK	81	AUR due to BPH, RV 500–1500 mL	TWOC day 2 + alfuzosin 5 mg ×2 TWOC day 2 + placebo ×2	22 12	40 41	55.0 29.3
Hastie 1990^87^	Prospective cohort study	UK	76	AUR	TWOC day 2	10	43	23.3

Abbreviations: AUR, acute urinary retention; BPH, benign prostate hyperplasia; RCT, randomized controlled trial; RV, residual volume; TWOC, trial without catheter.

^a^ Significance not tested in the study. Tested by us using an online calculator from Epitools.^93^

### 3.3 Risk of bias in studies

We had some concerns about risk of bias in the two RCTs (Figure S1). In both studies, Djavan 1998 and Taube 1987, this was due to a lack of description of the randomization process.^26^, ^27^ For the other domains, the risk of bias was considered low in both RCTs.

Among the comparative cohort studies (Figure S2), we considered Bouras 2018 at critical risk of bias.^28^ The study design was unclear, resulting in a critical risk of confounding and deviations from the seemingly intended interventions, and the outcome was only reported for 37 of the 77 participants. In the other two comparative cohort studies, Ko 2012 and Kim 2008, there were serious risks of bias.^29^, ^30^ In both studies, some possible confounding factors were not reported or adjusted for, both were retrospective with the resulting risk of selection bias, and the doctors' decision of immediate or delayed TWOC may have been influenced by the perceived prognosis. In Ko 2012, there was also a prospective part where the assignment to immediate or delayed TWOC was based on a prognostic tool developed for this purpose.^29^ This introduced a critical risk of selection bias in the prospective cohort.

The rest of the studies all reported on cohorts with either immediate or delayed TWOC without any comparison of the two strategies. Hence, there was a serious risk of bias due to confounding in all of them (Figures S3 and S4). In the retrospective studies, Breum 1982, Gas 2019, Park 2012, Rasner 2009, Tsui 2008 and Park 2006, as well as in the case–control study Tang 2015 where half the participants were included retrospectively, there was a serious risk of selection bias.^10^, ^18^, ^88^, ^89^, ^90^, ^91^, ^92^ The risk of selection bias was also serious in Klarskov 1987 where 55/228 participants were given permanent in‐dwelling catheters and in Green 2014 where 64/187 participants were selected directly for operative treatment and no TWOC.^20^, ^67^ The risk of perceived prognosis affecting the choice of intervention was also serious in these two studies, as well as in the retrospective part of Tang 2015 where inclusion was based on treatment with terazosin and in Gas 2019 where 153 patients were excluded due to hospitalization.^18^, ^20^, ^67^, ^92^ Considering our specified target study, co‐interventions were unbalanced across interventions when alpha‐blockers were given in the non‐comparative studies, yielding a serious risk of bias. There was also a serious risk of bias due to missing outcome data in Mahadik 2013, Pandit 2008, Tsui 2008 and Shah 2002.^52^, ^69^, ^74^, ^89^

### 3.4 Results of individual studies

In an RCT with 114 participants, Djavan 1998 (Table 1), success rates were 45% for immediate TWOC, 51% for TWOC after 2 days and 62% for TWOC after 7 days.^26^ No statistical testing was reported on this outcome. We calculated the relative success rate for delayed versus immediate TWOC to be 1.26 (95% CI 0.84–1.90).

In another RCT with 60 participants, Taube 1989 (Table 1), success rates were 28% for immediate TWOC, 20% for TWOC after 24 h and 36% for TWOC after 48 h, with no statistically significant differences.^27^ We calculated the relative success rate for delayed versus immediate TWOC to be 1.03 (95% CI 0.42–2.49).

In a prospective cohort study with outcome reported for 37 participants, Bouras 2018 (Table 1), success rates were 75% for immediate TWOC, 64% for TWOC after 2 days, 70% for TWOC after 3 days and 22% for TWOC after 10 days, with no statistically significant differences.^28^

In a retrospective study with 515 participants treated with tamsulosin 0.2 mg, Ko 2012 (Table 1), success rates were 25% for immediate TWOC and 30% for TWOC after 7 days, with no statistically significant difference (*p* = 0.71).^29^ In the prospective part of this study, where 28 participants treated with tamsulosin 0.2 mg were selected to immediate TWOC or TWOC after 7 days based on an algorithm developed from the retrospective study, success rates were 71% and 76%, respectively.^29^ For the retrospective part of the study, we calculated the relative success rate for delayed versus immediate TWOC to be 1.21 (95% CI 0.90–1.62).

In another retrospective study with 127 participants treated with tamsulosin 0.2 mg, Kim 2008 (Table 1), success rates were 48% for immediate TWOC and 55% for TWOC after 7 days.^30^ No statistical testing was reported on this outcome. We calculated the relative success rate for delayed versus immediate TWOC to be 1.14 (95% CI 0.82–1.60).

For the non‐comparative studies, the TWOC success rate for each separate cohort is reported in Table 1. Four studies with in total of 409 participants reported success rates for immediate TWOC in the range of 25%–88%.^10^, ^20^, ^31^, ^32^ Furthermore, 52 studies with in total 12 489 participants reported success rates for delayed TWOC in the range of 17%–80%.^12^, ^18^, ^19^, ^33^, ^34^, ^35^, ^37^, ^38^, ^39^, ^40^, ^41^, ^42^, ^43^, ^44^, ^46^, ^50^, ^51^, ^52^, ^53^, ^54^, ^55^, ^59^, ^60^, ^61^, ^62^, ^63^, ^64^, ^65^, ^66^, ^67^, ^68^, ^69^, ^72^, ^73^, ^74^, ^75^, ^76^, ^77^, ^78^, ^79^, ^80^, ^82^, ^83^, ^84^, ^85^, ^86^, ^87^, ^88^, ^89^, ^90^, ^91^, ^92^

Among the secondary outcomes, complications and adverse events were only reported in six studies (Table S4),^12^, ^33^, ^38^, ^39^, ^43^, ^46^ patient satisfaction and acceptability in four studies (Table S5)^50^, ^66^, ^78^, ^86^ and health care provider satisfaction and acceptability in none. Secondary outcomes were only reported in studies of delayed TWOC. However, comparing TWOC after 3 days or sooner to TWOC after more than 3 days, Fitzpatrick 2012 found adverse events in fewer patients undergoing TWOC early, 20% versus 34% (*p* < 0.001).^12^ Along the same lines, comparing TWOC after 3 or 7 days, Salem Mohamed 2018 found catheter complications in 17% versus 43% (*p* = 0.02).^33^

### 3.5 Results of syntheses

**FIGURE 2:**
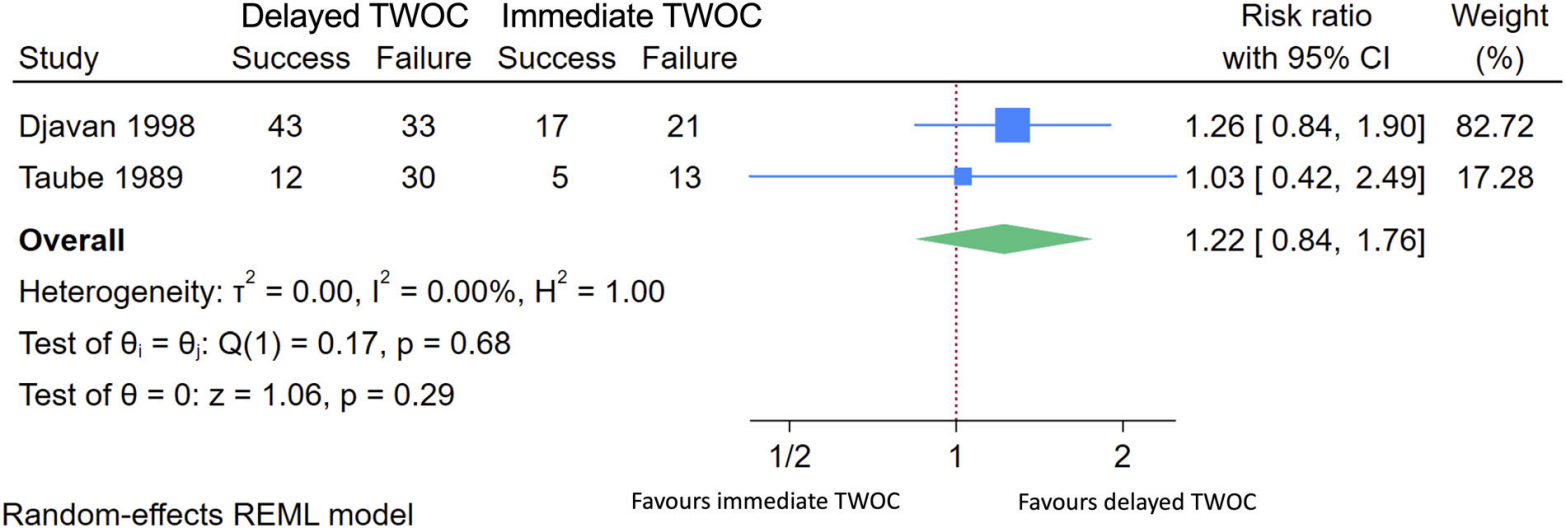
Meta‐analysis of RCTs comparing delayed and immediate TWOC.

In a meta‐analysis of the two RCTs, Djavan 1998 and Taube 1987, including in total of 174 participants, we found no statistically significant difference between the two TWOC strategies, relative success rate for delayed versus immediate TWOC 1.22 (95% CI 0.84–1.76, heterogeneity *I* ^2^ = 0%) (Figure 2).^26^, ^27^ In both RCTs, there were some concerns for bias related to the randomization process.

**FIGURE 3:**
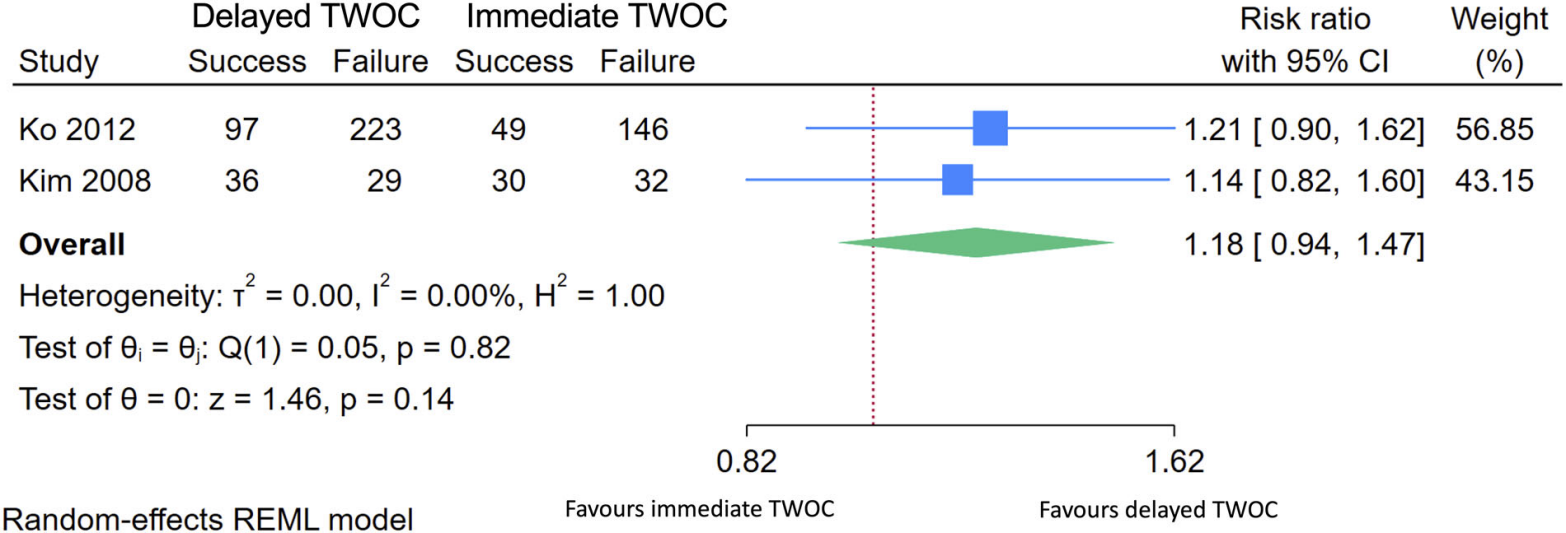
Meta‐analysis of comparative cohort studies comparing delayed and immediate TWOC.

In a meta‐analysis of the comparative cohort studies, we included Ko 2012 and Kim 2008, both with serious risk of bias.^29^, ^30^ We excluded the prospective part of Ko 2012, as we assessed the risk of bias critical in this cohort. Furthermore, we did not include Bouras 2018, also considered at critical risk of bias.^28^ The meta‐analysis comprised 642 participants and revealed no statistically significant difference between the two TWOC strategies, with a relative success rate for delayed versus immediate TWOC 1.18 (95% CI 0.94–1.47, heterogeneity *I* ^2^ = 0%) (Figure 3).

Four studies reported success rates for cohorts with immediate TWOC, all assessed as having a serious risk of bias.^10^, ^20^, ^31^, ^32^ They were all included in a meta‐analysis, yielding a total of 409 participants. The overall success rate for all the immediate TWOC cohorts was 47% (95% CI 29–66, heterogeneity *I* ^2^ = 90%) (Figure S5). In the cohorts not receiving alpha‐blocker, including 250 participants, the overall success rate was 33% (95% CI 19–48, heterogeneity *I* ^2^ = 73%). In the cohorts with alpha‐blocker, including 159 participants, the overall success rate was 66% (95% CI 41–88, heterogeneity *I* ^2^ = 63%).

Fifty‐two studies reported success rates for cohorts with delayed TWOC, all assessed as at serious risk of bias.^12^, ^18^, ^19^, ^33^, ^34^, ^35^, ^37^, ^38^, ^39^, ^40^, ^41^, ^42^, ^43^, ^44^, ^46^, ^50^, ^51^, ^52^, ^53^, ^54^, ^55^, ^59^, ^60^, ^61^, ^62^, ^63^, ^64^, ^65^, ^66^, ^67^, ^68^, ^69^, ^72^, ^73^, ^74^, ^75^, ^76^, ^77^, ^78^, ^79^, ^80^, ^82^, ^83^, ^84^, ^85^, ^86^, ^87^, ^88^, ^89^, ^90^, ^91^, ^92^ They were all included in a meta‐analysis, yielding a total of 12 489 participants. The overall success rate for all the delayed TWOC cohorts was 53% (95% CI 49–56, heterogeneity *I* ^2^ = 90%). In the cohorts not receiving alpha‐blocker, including 1294 participants, the overall success rate was 43% (95% CI 36–49, heterogeneity *I* ^2^ = 80%) (Figure S6). In the cohorts with alpha‐blocker, including 11 195 participants, the overall success rate was 57% (95% CI 53–60, heterogeneity *I* ^2^ = 90%) (Figure S7).

### 3.6 Reporting biases

In the two included RCTs, Djavan 1998 and Taube 1989, the risk of bias due to missing outcome data was assessed as low (Figure S1).^26^, ^27^ Also, in the two comparative studies included in the meta‐analysis, Ko 2012 and Kim 2008, the risk of bias due to missing outcome data was assessed as low (Figure S2).^29^, ^30^ However, there was a serious risk of bias due to missing outcome data in a minority of the studies included in the meta‐analyses of cohorts reporting success rates of delayed TWOC, Mahadik 2013, Pandit 2008, Tsui 2008 and Shah 2002 (Figure S4).^52^, ^69^, ^74^, ^89^

The usual direction of publication bias suggests that studies with a statistically significant result are more likely to be published than studies with statistically non‐significant findings. Our search identified two RCTs and three other studies comparing directly the two TWOC strategies of interest.^26^, ^27^, ^28^, ^29^, ^30^ None of them found any statistically significant difference in TWOC success rates. Therefore, we have no reason to believe that publication bias is a problem in this field.

### 3.7 Certainty of evidence

We assessed the certainty of the evidence from the randomized comparative studies as low, and from the non‐randomized comparative cohort studies as very low (Table 2). For our purposes, we also considered the certainty of the evidence from the non‐comparative studies as very low, as they did not involve direct comparisons and exhibited substantial inconsistency, with *I* ^2^ ranging from 63% to 90%.

**TABLE 2:** Summary of evidence.

No. of studies	Design	Risk of bias	Inconsistency	Indirectness	Imprecision	Other	Certainty (overall score)
Outcome: Success rate of delayed TWOC vs. immediate TWOC							
2	Randomized controlled trials	No serious risk	Not important *I* ^2^ = 0.0%	No serious indirectness	Very serious imprecision (low *n*, small effect estimate, wide 95% CI)	None	Low
Outcome: Success rate of delayed TWOC vs. immediate TWOC							
2	Comparative cohort studies	Serious risk	Not important *I* ^2^ = 0.0%	No serious indirectness	Serious imprecision (adequate *n*, small effect estimate, wide 95% CI)	None	Very low
Success rate of delayed TWOC vs. immediate TWOC						
People: Males ≥18 years of age catheterized for acute urinary retention Settings: Hospital urology and emergency departments in Austria, the UK and Korea Intervention: Delayed TWOC Comparison: Immediate TWOC							
Outcomes	Absolute Effect			Relative effect (95% CI)	Number of studies	Certainty of the evidence (GRADE)^a^	
	Delayed TWOC		Immediate TWOC				
Success rate in randomized controlled trials	47%		39%	1.22 (0.84–1.76)	2	_⊕⊕⊖⊖_ Low^c^	
Success rate in comparative cohort studies	35%		31%	1.18 (0.94–1.47)	2	_⊕⊖⊖⊖_ Very low^d^	

*Note*: High = This research provides a very good indication of the likely effect. The likelihood that the effect will be substantially different^b^ is low. Moderate = This research provides a good indication of the likely effect. The likelihood that the effect will be substantially different^b^ is moderate. Low = This research provides some indication of the likely effect. However, the likelihood that it will be substantially different^b^ is high. Very low = This research does not provide a reliable indication of the likely effect. The likelihood that the effect will be substantially different^b^ is very high. 95% CI: 95% confidence interval.

^a^ GRADE Working Group grades of evidence.

^b^ Substantially different = a large enough difference that it might affect a decision.

^c^ Starting at high confidence, rated down two levels for very serious imprecision.

^d^ Starting at low confidence (serious risk of bias), rated down one level for serious imprecision.

## 4 DISCUSSION

We did not find any statistically significant difference in success rates between immediate and delayed TWOC in the two RCTs or the two other comparative studies. Hence, there is no support in the evidence to prefer one TWOC strategy over the other. However, in the non‐comparative studies, there was a somewhat higher overall success rate for delayed compared with immediate TWOC, with success rates of 53% versus 47%, respectively. It is important to note that these latter results are of very low certainty.

The high success rates for delayed TWOC were mainly seen in the cohorts where an alpha‐blocker was given. The beneficial effect of alpha‐blockers in this situation is previously well documented.^5^, ^13^ Though one might expect that the onset of the effect of alpha‐blockers was not fast enough to impact on the success of immediate TWOC, Chan 1996 reported significantly higher success rates when immediate TWOC was accompanied by terazosin.^31^ Still, alpha‐blockers did not affect our main result that there was no difference between the strategies, as no patients received alpha‐blockers in the two RCTs, and all patients received alpha‐blockers in the two other comparative studies.

Ko 2012 developed an algorithm based on retrospective data, where larger retention volume and higher age pointed in the direction of delayed TWOC, increasing the success rate for both strategies in a small validating study.^29^ Several studies have found smaller retention volumes to be associated with successful immediate TWOC,^20^, ^26^, ^30^, ^32^ and Klarskov 1987 also found a higher immediate TWOC success rate for precipitated urinary retention.^20^ These associations have also been shown for delayed TWOC.^12^

We were unable to compare the two strategies regarding the rate of complications, adverse events and patient and health care provider satisfaction and acceptability, as the secondary outcomes were only reported in studies of delayed TWOC, if at all. However, adverse events were more frequent when the catheter was left in place for more than 3 days,^12^, ^33^ suggesting that time before delayed TWOC should be kept short. Furthermore, though tolerating the catheter, many men find it cumbersome.^14^, ^15^

### 4.1 Strengths and limitations

We are not aware of any other systematic review addressing our research question. To comprehensively chart the field, we chose to use wide inclusion criteria, not only including studies comparing the two TWOC strategies directly.

We did not find any statistically significant difference between the success rates of the two TWOC strategies, but our meta‐analysis of the two RCTs with a total of 174 participants was highly likely underpowered to detect a difference.^26^, ^27^ Using an online sample size calculator from EpiTools,^93^ a total sample of 214 would be necessary to detect a difference between success rates of 40% and 60% with 80% probability.

In the retrospective design of the non‐randomized comparative studies, Ko 2012 and Kim 2008, the doctor decided the TWOC strategy at the time of catheterization, probably choosing immediate TWOC when it seemed most likely to succeed.^29^, ^30^ Consequently, these studies may overestimate the success rate of immediate TWOC compared with an unselected population, while underestimating the success rate of delayed TWOC, as this option probably was chosen for patients presumably less responsive to immediate treatment.

Though there was no standard definition of successful TWOC (Table S2), we consider the ability to void successfully a rather obvious and sufficiently clear clinical outcome to justify combining the studies in the meta‐analyses.

The search was extensive in that we had no restrictions on language or publication date. All the preliminarily identified studies were found in the search. Furthermore, we searched the reference lists in all included studies and in the background literature used in this paper.

We included studies from all over the world, which strengthens the generalizability of our results. Though there may be some imprecision in the translation when using Google Translate, we minimized this concern by having two researchers independently extracting data and assessing risk of bias.

### 4.2 Implications

Low‐certainty evidence showed no statistically significant difference in the success rate of the two TWOC strategies. However, the meta‐analysis of the two RCTs was underpowered. Hence, the next research step should be a sufficiently powered RCT. Alpha‐blockers have been shown to be beneficial for successful delayed TWOC.^13^ There are indications that this might also apply to immediate TWOC.^31^ This question should be investigated further, preferably in a sufficiently powered randomized study comparing immediate TWOC with and without alpha‐blocker to delayed TWOC with alpha‐blocker. It would not be appropriate to include an arm of delayed TWOC without alpha‐blocker, as this has already been shown to be inferior.^13^ Another approach could be to develop criteria for deciding which TWOC strategy to recommend, along the lines of the algorithm developed by Ko 2012.^29^

While awaiting the results of a future sufficiently powered RCT or reliable criteria for choosing TWOC strategy, there seems to be a somewhat higher success rate for delayed compared with immediate TWOC. Still, the difference is small, and the chance of successful immediate TWOC seems large enough that many men with acute urinary retention would give it a try to avoid the inconvenience and complications associated with using a catheter, as long as they can easily access an emergency service in case of TWOC failure. We suggest offering patients the choice of TWOC strategy, providing them with our best estimates for the success of each.

## AUTHOR CONTRIBUTIONS

Odd Martin Vallersnes conceived the study. Veronika S. Christensen, Marius Skow, Signe A. Flottorp, Hilde Strømme and Odd Martin Vallersnes designed the study. Hilde Strømme performed the literature search. Veronika S. Christensen, Marius Skow and Odd Martin Vallersnes screened the retrieved records, reviewed full‐text reports, selected studies for inclusion and extracted data. Marius Skow, Signe A. Flottorp and Odd Martin Vallersnes assessed the risk of bias in the studies. Ibrahimu Mdala performed the meta‐analyses. Signe A. Flottorp and Odd Martin Vallersnes assessed the certainty of the evidence. Veronika S. Christensen and Odd Martin Vallersnes drafted the manuscript. All authors reviewed the manuscript and approved the final version.

## CONFLICT OF INTEREST STATEMENT

The authors report no conflict of interests.

## Supporting information


**Table S1.** Search strategies.

**Table S2.** Study funding, setting, TWOC success definition, and intervention details.

**Table S3.** Inclusion/exclusion criteria and patient characteristics.

**Table S4.** Studies reporting secondary outcomes – complications and adverse events.

**Table S5.** Studies reporting secondary outcomes – patient satisfaction.

**Figure S1.** Risk of bias assessment in the included randomized controlled trials, performed in RoB 2.0.^21^

**Figure S2.** Risk of bias assessment in the included comparative cohort studies, performed in ROBINS‐I.^22^

**Figure S3.** Risk of bias assessment in the included cohort studies reporting only immediate TWOC, performed in ROBINS‐I.^22^

**Figure S4.** Risk of bias assessment in the included cohort studies reporting only delayed TWOC, performed in ROBINS‐I.^22^

**Figure S5.** Meta‐analysis of cohort studies reporting success rates for immediate TWOC.

**Figure S6.** Meta‐analysis of cohort studies reporting success rates for delayed TWOC for patients not given alpha‐blockers.

**Figure S7.** Meta‐analysis of cohort studies reporting success rates for delayed TWOC for patients given alpha‐blockers.

## Data Availability

Data materials not published in the review are available on reasonable request to the corresponding author.
