# Supplementary material for: Immediate or delayed trial without catheter in acute urinary retention in males: A systematic review
Source: BJUI Compass. 2024 May 14;5(8):732–47. doi: 10.1002/bco2.369 (PMC11327489; doi:10.1002/bco2.369)
Supplement: Supplementary file 1 — Table S1. Search strategies. [file BCO2-5-732-s003.pdf]

Supplementary table 1. Search strategies

**Ovid MEDLINE(R) ALL <1946 to November 29, 2022>**		**Records**
1	Male/	9 287 405
2	(man or men or male or males).tw,kf.	2 108 373
3	Urinary Retention/	5 058
4	((urin* adj2 retention) or ischuria*).tw,kf.	11 929
5	Urinary Catheterization/	14 645
6	(catheteri* adj1 (urin* or uret* or foley or bladder)).tw,kf.	4 281
7	(1 or 2) and (3 or 4) and (5 or 6)	913
8	7 not (exp "Animals"/ not (exp "Animals"/ and "Humans"/))	901
9	8 not (Editorial or Letter).pt.	876
**Embase Classic+Embase <1947 to 2022 November 29>**		**Records**
1	Male/	11 235 812
2	(man or men or male or males).tw,kf.	3 262 908
3	urine retention/	33 979
4	((urin* adj2 retention) or ischuria*).tw,kf.	21 307
5	bladder catheterization/ or urethral catheterization/	10 206
6	(catheteri* adj1 (urin* or uret* or foley or bladder)).tw,kf.	6 490
7	(1 or 2) and (3 or 4) and (5 or 6)	1 415
8	7 not ("animal"/ not "human"/)	1 415
9	8 not (Editorial or Letter).pt.	1 356
**Cochrane Database of Systematic Reviews (Cochrane Library, Wiley)**		**Records**
**Cochrane Central Register of Controlled Trials (Cochrane Library, Wiley)**		
#1	[mh ^Male]	462 168
#2	(man OR men OR male OR males):ti,ab,kw	839 219
#3	[mh ^"Urinary Retention"]	460
#4	((urin* NEAR/2 retention) OR ischuria*):ti,ab,kw	3 944
#5	[mh ^"Urinary Catheterization"]	760
#6	(catheteri* NEAR/1 (urin* OR uret* OR foley OR bladder)):ti,ab,kw	1 515
#7	(#1 OR #2) AND (#3 OR #4) AND (#5 OR #6) in Cochrane Reviews, Cochrane Protocols	6
#8	(#1 OR #2) AND (#3 OR #4) AND (#5 OR #6) in Trials	159
**OpenGrey**		**Records**
	((man OR men OR male OR males) AND ("urinary retention" OR "urine retention" OR ischuria*) AND catheteri*)	0
**ClinicalTrials.gov**		**Records**
	Condition or disease: ("urinary retention" OR "urine retention" OR ischuria*)<br>AND<br>Other terms: (man OR men OR male OR males) AND catheterization	28

Search performed 30 November 2022, except OpenGrey which was searched on 11 May 2021.
