# Supplementary material for: Immediate or delayed trial without catheter in acute urinary retention in males: A systematic review
Source: BJUI Compass. 2024 May 14;5(8):732–47. doi: 10.1002/bco2.369 (PMC11327489; doi:10.1002/bco2.369)
Supplement: Supplementary file 2 — Table S2. Study funding, setting, TWOC success definition, and intervention details. [file BCO2-5-732-s004.pdf]

Supplementary table 2. Study funding, setting, TWOC success definition, and intervention details

Study	Funding	Setting	Successful TWOC definition	Intervention details
**RCTs comparing immediate TWOC with delayed TWOC**				
Djavan 1998 *[26]*	NR	Hospital	Ability to void after catheter removal	Immediate TWOC<br>TWOC day 2<br>TWOC day 7
Taube 1989 *[27]*	NR	Hospital urology department	Ability to establish satisfactory micturition after catheter removal	Immediate TWOC<br>TWOC 24 hours<br>TWOC 48 hours
**Other studies comparing immediate TWOC with delayed TWOC**				
Bouras 2018 *[28]*	NR	Hospital urology department	No AUR within 24 hours of ablation	Immediate TWOC<br>TWOC day 2<br>TWOC day 3<br>TWOC day 10
Ko 2012 *[29]*	NR	University hospital urology department	Successful self-voiding with max RV < 150 mL and no need for catheterization the initial 7 days after catheter removal	Immediate TWOC + tamsulosin 0.2 mg<br>TWOC day 7 (mean day 6.6) + tamsulosin 0.2 mg
Kim 2008 *[30]*	NR	Hospital urology department and ED	Ability to void after removal of catheter and no re-catheterization the next 24 hours	Immediate TWOC + tamsulosin 0.2 mg<br>TWOC day 7 + tamsulosin 0.2 mg

**Studies reporting success rate of immediate TWOC**				
Li 2009 *[32]*	NR	Hospital ED	Successful micturition without re-catheterization within 24 hours of catheter removal	Immediate TWOC (all included patients)
Chan 1996 *[31]*	NR	Hospital	Urination after removal of catheter	Immediate TWOC + terazosin 10 mg<br>Immediate TWOC + terazosin 5 mg<br>Immediate TWOC + placebo<br>Immediate TWOC = 2 hours after catheterization and medication
Klarskov 1987 *[20]*	NR	Hospital urology departments	No recurrent retention within a week	Immediate TWOC
Breum 1982 *[10]*	NR	Hospital ED	No recurrent retention within a week	Immediate TWOC
**Studies reporting success rate of delayed TWOC**				
Khadka 2021 *[60]*	NR	Teaching hospital	Ability to pass > 100 mL urine within 6 hours of catheter removal, with < 200 mL post void residual volume, and not needing re-catheterization within 24 hours	TWOC day 7 + alfuzosin 10 mg
Phuong Hoai 2021 *[61]*	None	University hospital	Ability to pass urine with < 100 mL post void residual volume, and not needing re-catheterization within 24 hours	TWOC day 3 + alfuzosin 10 mg
Jha 2020 *[62]*	None	Hospital urology department	Ability to pass > 200 mL of urine within 6 hours of catheter removal with a max flow rate of 5 mL/s on uroflowmetry and achieved a postvoid RV < 150 mL on trans-abdominal ultrasound	TWOC day 3 + tamsulosin 0.4 mg

Gas 2019 *[18]*	NR	University hospital urology department and ED	Ability to void after catheter removal	TWOC day 18 (mean 17.5) + alpha-blocker (given to 90%)
Kurniasari 2019 *[79]*	None	Hospital urology department	Spontaneous micturition < 6 hours after catheter withdrawal, post-void RV < 100 mL, and uroflowmetry Qmax > 10 mL/s	TWOC day 5
Vella 2019 *[63]*	NR	Tertiary hospital urology department and ED	Spontaneous micturition with RV < 300 mL	TWOC day 14 + alpha-blocker + fluoroquinolone + Serenoa repens extract
Das 2018 *[64]*	NR	Hospital urology department	Voiding within 6 hours with a max flow rate > 5 mL/s and RV < 200 mL	TWOC day 4 + tamsulosin 0.4 mg
Salem Mohamed 2018 *[33]*	None	Hospital urology departments (2 centres)	Ability to pass > 200 mL of urine after catheter removal	TWOC day 3 + tamsulosin 0.4 mg + levofloxacin 500 mg<br>TWOC day 7 + tamsulosin 0.4 mg + levofloxacin 500 mg
Bansal 2017 *[19]*	None	Hospital	Voiding with RV < 100 mL and no need for re-catheterization next 24 hours	TWOC day 4 (mean 4.3, range 3–7) + alpha-blocker (alfuzosin 10 mg 36.2%, tamsulosin 0.4 mg 41.7%, silodosin 8 mg 22.1%)
Farelo-Trejos 2017 *[65]*	NR	Hospital urology ED	Spontaneous micturition immediately and up to 20 days after removal of catheter	TWOC day 10 + tamsulosin 0.4 mg
Ferdian 2016 *[80]*<br><br>*Jouwena 2016 [81]*	NR	University hospital urology department	Voiding well after removal of catheter	TWOC "after a certain period"
Hagiwara 2016 *[66]*	NR	University hospital urology department	Ability to void voluntarily with a postvoid RV of < 100 mL and not needing recatheterization the next 24 hours	TWOC day 14 + silodosin 4 mg x2 + dutasteride 0.5 mg (primary endpoint cumulative successful TWOC at 12 weeks)

Tang 2015 *[92]*	NR	Hospital ED	Successful self-voiding after removal of catheter and no retention requiring catheterization within 3 days	TWOC day 4 (mean 4.5 days, range 3–7) + alfuzosin 10mg<br>TWOC day 4 (mean 4.2 days, range 3–7) + terazosin 2-4 mg (1 mg 1.7%, 2 mg 32.3%, 4 mg 62.7%, 5-6mg 3.4%)
Green 2014 *[67]*	NR	Hospital urology department	Voiding with RV < 200 mL on 3 successive occasions after removal of catheter	TWOC day 13 (mean) + alpha-blocker
Kara 2014 *[34]*	NR	University hospital	Ability to empty bladder and not requiring recatheterization on the day of catheter removal	TWOC day 3 + tamsulosin 0.4 mg<br>TWOC day 3 + tamsulosin 0.4 mg + alfuzosin 10 mg
Maldonado-Ávila 2014 *[35]*<br><br>*Maldonado-Ávila 2012 [36]*	NR	Hospital outpatient service	Successful voiding of > 100 mL and RV < 200 mL	TWOC day 5 (mean day 12.7) + tamsulosin 0.4 mg<br>TWOC day 5 (mean day 9.7) + alfuzosin 10 mg<br>TWOC day 5 (mean day 14.3) + placebo
Sharifi 2014 *[37]*	NR	Hospital urology department	Successful voiding 7 days following catheter removal	TWOC day 3 + tamsulosin 0.4 mg + sildenafil 50 mg<br>TWOC day 3 + tamsulosin 0.4 mg + placebo
Zhengyong 2014 *[38]*	NR	Hospital urology department and ED	Voiding with RV < 150 mL and no need for re-catheterization the next 24 hours	TWOC day 7 + tamsulosin 0.2 mg + finasteride 5 mg + bladder training<br>TWOC day 7 + tamsulosin 0.2 mg + finasteride 5 mg + free drainage
Elbendary 2013 *[39]*	NR	University hospital urology department	Successful voiding after removal of catheter (none had a new AUR the following month)	TWOC day 7 + tamsulosin 0.4 mg + ketoconazole 200 mg<br>TWOC day 7 + tamsulosin 0.4 mg + placebo
Kumar 2013 *[40]*	NR	Tertiary hospital urology department	Voiding successfully > 100 mL with RV < 150 mL and not re-experiencing painful AUR within 2 weeks	TWOC day 3 + silodosin 8 mg<br>TWOC day 3 + placebo
Lodh 2013 *[68]*	NR	Hospital	Ability to pass urine with post-void RV ≤ 150 mL and max flow rate ≥ 10 mL/s	TWOC day 8 + tamsulosin 0.4 mg

Mahadik 2013 *[69]*	NR	Hospital urology outpatient clinic	Voiding > 100 mL within 6 hours of catheter removal with RV < 200 mL	TWOC day 2 + tamsulosin 0.4 mg
Sharis 2013 *[82]*	None	University hospital urology department	Voiding with RV < 150 mL and uroflowmetry max flow rate 10 mL/s	TWOC day 10
Fitzpatrick 2012 *[12]*<br><br>*Emberton 2008 [70]*<br><br>*Desgrandchamps 2006 [71]*	Sanofi	Urologists in public, private, or mixed health care services	Successful voiding	TWOC day 5 (median; 1.8% immediate TWOC but outcome not reported separately) + alpha-blocker (given to 85.9%)
Park 2012 *[91]*	NR	University hospital urology department	Voiding successfully	TWOC day 9<br>30 included patients had immediate surgery, hence not TWOC
Bhomi 2011 *[72]*	NR	Teaching hospital surgery department	Voiding with RV < 150 mL and no need for re-catheterization next 24 hours	TWOC day 3 + tamsulosin 0.4 mg
Zeif 2010 *[83]*	NR	Hospital urology department	Successful voiding	TWOC day 1 (catheterization on admission, subsequent TWOC, with or without alpha-blocker (not recorded))
Agrawal 2009 *[41]*	None	Hospital urology department	Succesful voiding after removal of catheter	TWOC day 3 + alfuzosin 10 mg<br>TWOC day 3 + tamsulosin 0.4 mg<br>TWOC day 3 + placebo
Daly 2009 *[73]*	NR	University hospital ED and urology department	Successful voiding	TWOC day 28 + alpha-blocker (a variety was used, at the discretion of the ED doctor)
Rasner 2009 *[88]*	NR	Hospital urology department	Recovery of physiological voiding	TWOC day 4 + alfuzosin 10 mg + tamsulosin 0.4 mg<br>TWOC day 4 + alfuzosin 10 mg

Tiong 2009 *[42]*	NR	Hospital urology department	Voiding ≥ 100 mL with RV < 150 mL	TWOC day 2 + alfuzosin 10 mg<br>TWOC day 2 + placebo<br>Trial medication started at inclusion; TWOC at least 2 hours after second dose of trial medication
Panda 2008 *[84]*	NR	-	Successful voiding	TWOC day 3
Pandit 2008 *[74]*	NR	Hospital	No recurrent AUR within a week	TWOC day 9 (range 3–15) + alpha-blocker (“most patients” given terazosin, prazosin, or tamsulosin)
Tsui 2008 *[89]*	NR	Hospital ED	Successful voiding assessed by urologist	TWOC day 3 + terazosin 2 mg<br>TWOC day 3 + terazosin 4 mg
Al-Hashimi 2007 *[43]*	NR	Hospital	Ability to void within 24 hours of catheter removal with a flow rate > 5 mL/s, a voided volume > 100 mL and RV < 200 ml	TWOC day 3 + alfuzosin 10 mg<br>TWOC day 3 + placebo
Mariappan 2007 *[75]*	NR	Hospital urology department	Ability to void ≥ 50% of pre-voiding volume	TWOC day 14 + alfuzosin 10 mg
Gopi 2006 *[76]*	NR	Hospital ED	Ability to void after removal of catheter	TWOC day 6 (range 5–7) + alfuzosin 10 mg
Park 2006 *[90]*	NR	Hospital urology departments and EDs	Ability to urinate after catheter removal	TWOC day 7 + alpha-blocker
Lucas 2005 *[44]*<br><br>*Lucas 2002 [45]*	Yamanouchi Pharma Ltd	Hospital urology departments	Successful voiding of > 100 mL with RV < 200 mL and flow rate > 5 mL/s	TWOC day 5 (range 3–8) + tamsulosin 0.4 mg<br>TWOC day 5 (range 3–8) + placebo
Lorente Garín 2004 *[50]*	NR	Hospital urology department	Spontaneous voiding	TWOC day 7 + doxazosin 4 mg<br>TWOC day 7

McNeill 2004 *[46]*<br><br>*McNeill 2005 [47]*<br><br>*Hargreave 2003 [48]*<br><br>*McNeill 2003 [49]*	Sanofi-Aventis<br>Sanofi-Synthelabo	Hospital urology departments	Satisfactory voiding within 24 hours following removal of catheter without re-catheterization	TWOC day 2 (mean 53 hours) + alfuzosin 10 mg<br>TWOC day 2 (mean 53 hours) + placebo
Hua 2003 *[51]*	NR	University hospital urology department	Ability to urinate spontaneously	TWOC day 3 + tamsulosin 0.4 mg<br>TWOC day 3
Tan 2003 *[85]*	Singapore National Medical Research Council	Hospital urology department	Satisfactory micturition: post-void RV <100 mL and max flow rate >10 mL/s	TWOC day 2
Shah 2002 *[52]*	Lorex Synthelabo Pharma	Hospital	Ability to void with RV <200 mL	TWOC day 2 + alfuzosin 5 mg x2<br>TWOC day 2 + placebo x2<br>(Day 2 = after a minimum of 3 doses of medication or 36 hours after admission)
Abeygunasekera 2001 *[77]*	NR	Teaching hospital urology unit	Successful voiding after removal of catheter	TWOC day 7 + prazosin
Bowden 2001 *[53]*	NR	Hospital	Ability to void after removal of catheter	TWOC day 2 + tamsulosin 0.4 mg<br>TWOC day 2 + placebo
Kim 2001 *[78]*	NR	University hospital urology department	Ability to void adequately	TWOC day 7 (mean 6.8, range 4–21) + tamsulosin 0.4 mg
Perepanova 2001 *[54]*	NR	Urological hospital	Persistent urination for 3 days after removal of catheter	TWOC day 1 + doxazosin 4 mg<br>TWOC day 1 + placebo<br>Day 1 = after 12 hours

Kumar 2000 *[86]*	NR	Hospital urology department	Voiding successfully with postvoid RV ≤200 mL	TWOC day 2 or when any constipation was cleared
Lim 1999 *[59]*	NR	Hospital urology department	Ability to re-establish satisfactory micturition without a palpable bladder	TWOC day 1 (within 24 hours)<br>TWOC day 2 (after 24 hours)
McNeill 1999 *[55]*<br><br>*McNeill 2004 [56]*<br><br>*McNeill 1998 [57]*<br><br>*McNeill 2000 [58]*	Lorex Synthélabo	Hospital	Return to satisfactory voiding and re-catheterization not required within 24 hours	TWOC day 2 (mean 42 hours) + alfuzosin 5 mg x2<br>TWOC day 2 (mean 42 hours) + placebo x2
Hastie 1990 *[87]*	NR	Hospital urology department	Voiding satisfactorily<br>Outcome reported for the 43 patients with transurethral catheter (not the 33 patients with suprapubic catheter)	TWOC day 2

AUR: acute urinary retention; ED: emergency department; NR: not reported; RCT: randomized controlled trial; RV: residual volume; TWOC: trial without catheter.
