# Supplementary material for: Immediate or delayed trial without catheter in acute urinary retention in males: A systematic review
Source: BJUI Compass. 2024 May 14;5(8):732–47. doi: 10.1002/bco2.369 (PMC11327489; doi:10.1002/bco2.369)
Supplement: Supplementary file 3 — Table S3. Inclusion/exclusion criteria and patient characteristics. [file BCO2-5-732-s007.pdf]

## Supplementary table 3. Inclusion/exclusion criteria and patient characteristics

| Study | Inclusion criteria | Exclusion criteria | Patient characteristics |
|---|---|---|---|
| **RCTs comparing immediate TWOC with delayed TWOC** | | | |
| ***Djavan 1998*** *[26]* | AUR | - | Age 72.3 years<br>RV 1171 mL |
| ***Taube 1989*** *[27]* | AUR (sudden, painful cessation of micturition) | Creatinine > 200mL; clot retention | Age 75.5 years<br>Hesitancy 58.3%<br>Nocturia x ≥ 3 46.7%<br>UTI 15.0%<br>RV 989 mL |
| **Other studies comparing immediate TWOC with delayed TWOC** | | | |
| ***Bouras 2018*** *[28]* | AUR | - | Age 70 years<br>History of BPH 76.2%<br>Alpha-blocker at presentation 60.1%, additional 16.8% started<br>Previous AUR 57.3%<br>LUTS 35.0%<br>Faecal impaction 9.8%<br>Positive urine culture 4.9%<br>RV 580 mL |
| ***Ko 2012*** *[29]* | BPH-related AUR (painful inability to void, RV ≤ 1500 mL, clinically benign prostate) | Previous prostate or urethral surgery; prostate cancer; spinal cord injury; urodynamic diagnosis of neurogenic bladder; lower urinary tract trauma; febrile symptoms suspicious of prostatitis | - |
| ***Kim 2008*** *[30]* | AUR (> 50 years of age) | Prostate cancer; urethral strictures; prostate or urethral surgery, epineuritic bladder; fever; patients requiring hospital admission | Age 73 years (median)<br>RV 900 mL (median)<br>Previous AUR 36.8% |

| | | | |
|---|---|---|---|
| **Studies reporting success rate of immediate TWOC** | | | |
| ***Li 2009*** *[32]* | AUR (≥ 40 years of age) | Gross haematuria; prostate carcinoma (known or suspected); urethral stricture; bladder stone; bed-ridden or wheel-chair bound patient, comorbidity necessitating hospitalisation | Age 70 years<br>History of BPH 76.2%<br>Alpha-blocker at presentation 60.1%, additional 16.8% started<br>Previous AUR 57.3%<br>LUTS 35.0%<br>Faecal impaction 9.8%<br>Positive urine culture 4.9%<br>RV 580 mL |
| ***Chan 1996*** *[31]* | AUR | - | - |
| ***Klarskov 1987*** *[20]* | AUR | - | Age 73 years (median)<br>RV 900 mL (median)<br>Previous AUR 36.8% |
| ***Breum 1982*** *[10]* | AUR due to diagnosed or assumed BPH, confirmed by mechanical emptying of ≥ 100 mL urine | Prostatic carcinoma; urethral stricture; other specific diagnosis than BPH | Age 70 years<br>RV 755 mL<br>Previous AUR 21.4% |

| | | | |
|---|---|---|---|
| **Studies reporting success rate of delayed TWOC** | | | |
| ***Khadka 2021*** *[60]* | AUR due to BPH (≥ 40 years of age) | Chronic retention, acute prostatitis, prostatic abscess or prostatic cancer and urethral stricture, recent surgery (<15 days), obstructive uropathy, history of myelopathy or polyneuropathy, patients taking anticholinergic drugs or antidepressants, patients coming before 7 days or failed to follow up after catheterization | Age 54–80 years (range)<br>Prostate size ≥ 50 mL 43.3%<br>AUA grade severe 56.7%<br>IPP grade III 26.7% |
| ***Phuong Hoai 2021*** *[61]* | AUR associated to BPH | Prostate cancer, prostatitis, urethral injury, pelvic fracture, urethral gravel, urethral stricture, bladder neck sclerosis, tabes dorsalis, spinal cord injury, spinal cord inflammation, or drug induced AUR (e.g. pseudoephedrine and antihistamines) | Age 75.0 years<br>IPSS 16.7<br>Prostate size 57.8 mL<br>RV 1000 mL<br>Duration of LUTS 1.8 years<br>Previous AUR 56.2%<br>Prostatic pain during DRE 16.4%<br>Urea 5.82 mmol/L<br>Creatinine 89.2 mmol/L<br>PSA 18.2 ng/dL<br>IPP 11.2 mm<br>UTI 50.0% (15/30) |
| ***Jha 2020*** *[62]* | First episode AUR secondary to BPH (age ≥ 45 years) | Already on alpha blocker, 5-alpha reductase inhibitor and/or anti-cholinergics drugs, RV > 1000 mL, suspicious digital rectal examination, urethral stricture, urethra/vesicle calculus, prior lower urinary tract surgery, neurological/psychiatric disorders, recent surgery/UTI/gross haematuria/perineal trauma | Age 66.8 years<br>IPSS 14.8<br>Prostate size 45.8 g<br>RV 688 mL<br>Duration of LUTS 7.1 months<br>BWT 5.8 mm<br>IPP > 10 mm 28.9% |
| ***Gas 2019*** *[18]* | AUR outpatient treatment | Patients requiring hospitalisation | Age 70 years<br>RV 992 mL<br>BPH 41.5%<br>Previous AUR 18.5%<br>On alpha-blocker 30.2%<br>Neurological disease 22.6% |

| | | | |
|---|---|---|---|
| ***Kurniasari 2019*** *[79]* | AUR due to BPH (> 50 years of age) | Recurrent AUR; previous prostate surgery; alpha-blocker therapy; 5-alpha-reductase inhibitor last 2 weeks; bladder stone; urethra stone; prostate malignancy; bladder malignancy; urethral stricture; diabetes; neurological disorders; UTI | Age 65 years<br>LUTS duration 12 months<br>RV 1054 mL<br>PSA 3.3 ng/mL<br>Prostate size 49 mL<br>DWT 2.3 mm<br>IPP 10.3 mm |
| ***Vella 2019*** *[63]* | AUR | Age < 40 years; febrile acute prostatitis; prostatic abscess; surgery last 15 days; obstructive uropathy (bilateral hydroureteronephrosis and creatinine > 2.5 mg/dL); myelopathy; polyneuropathy; urethral stone; massive fecal impaction; prostate cancer; anticholinergic drugs; antidepressants; Mb Parkinson; dementia | Age 68 years<br>IPSS 16<br>Prostate size 43 mL<br>RV 709 mL<br>PSA 2.8 ng/mL |
| ***Das 2018*** *[64]* | AUR due to BPH (clinical diagnosis) | Backpressure changes; previous failure of TWOC; recurrent haematuria; recurrent UTI; neurological illness; recent use of 5-alpha-reductase inhibitors, tricyclic antidepressants, anticholinergics, sympatomimetics, or first-generation antihistamines | Age 65 years<br>LUTS duration 8.3 months<br>RV 807 mL<br>Prostate size 59 mL<br>DWT ≥ 5 mm 87.8%<br>IPP > 10 mm 24.4% |
| ***Salem Mohamed 2018*** *[33]* | First episode AUR due to BPH | RV > 1000mL, bladder stones, renal impairment, suspected urethral stricture, neurogenic bladder, cancer prostate, medically induced retention, previous use of alpha-blockers, hypersensitivity or allergy to tamsulosin, chronic retention | Age 59.5 years<br>IPSS 13.3<br>Prostate size 59.7 mL<br>RV 673 mL<br>PSA 2.8 ng/mL |
| ***Bansal 2017*** *[19]* | First episode AUR due to BPH | Bladder stone; renal insufficiency due to bladder outlet obstruction; neurological impairment; need for suprapubic catheterization; gross haematuria; UTI; anticholinergics; previous urological surgery; uncontrolled diabetes or hypertension | Age 66 years<br>LUTS duration 14.6 months<br>AUA symptom score 19.7<br>Prostate size 56 mL<br>RV 781 mL<br>IPP 10 mm<br>PSA 3.9 ng/mL |

| | | | |
|---|---|---|---|
| ***Farelo-Trejos 2017*** *[65]* | AUR due to BPH (> 50 years of age), catheterized without complication | Urethral stricture; haematuria; clots; bladder lithiasis; prostate cancer; anaesthetic procedure from major surgery or post-operative surgery; lower urinary tract surgery; severe or unstable heart failure; cholinergics; anticholinergics; MAO inhibitors; severe liver failure | Age 68 years<br>Prostate size 100 g<br>PSA 4 ng/mL (median)<br>IPSS 17 (median)<br>Diabetes 18.5% |
| ***Ferdian 2016*** *[80]*<br><br>*Jouwena 2016 [81]* | First episode of AUR due to BPH | - | Age 64.5 years<br>Prostate size 53.5 g<br>IPSS 20.6 |
| ***Hagiwara 2016*** *[66]* | First episode of spontaneous AUR | UTI, urological tumors, clot retention, urethral stricture, chronic urinary retention, urolithiasis, drug abuse, neurogenic lower urinary tract dysfunction, history of prostatic surgery or urological treatment | Age 75 years (median)<br>IPSS 26 (median)<br>Prostate size 46.4 mL (median)<br>RV 500 mL (median)<br>PSA 5.80 ng/mL (median) |
| ***Tang 2015*** *[92]* | AUR due to presumed BPH (> 18 years of age) with RV ≥ 300 mL | Causes of AUR other than BPH e.g., urethral disease or spinal cord compression; significant obstructive uropathy; active urosepsis; potent CYP34A-inhibitors; moderate or severe hepatic impairment; allergy to alpha-blockers, postural hypotension; already on alpha-blockers | Age 72 years<br>Prostate size ≥ 3 finger breadths 72.4%<br>RV 600 mL (median)<br>Previous LUTS 57.8%<br>Anticholinergic medication 31.9%<br>UTI 15.5%<br>Faecal impaction 20.7%<br>Creatinine 86 umol/L (median) |
| ***Green 2014*** *[67]* | AUR | - | Age 71 years<br>RV 1078 mL<br>LUTS 75% |
| ***Kara 2014*** *[34]* | AUR due to BPH | RV > 1500 or < 500 mL, renal or hepatic dysfunction, previous urinary tract surgery, neurogenic or other diseases of the bladder, upper urinary tract diseases such as uremia, any malignancy, or the use of retention-enhancing medications | Age 71.2 years<br>RV 698 mL |

| | | | |
|---|---|---|---|
| ***Maldonado-Ávila 2014*** *[35]*<br><br>*Maldonado-Ávila 2012 [36]* | First episode AUR (> 50 years of age) | Previous AUR; treatment for prostatic growth; creatinine > 120 mmol/L; reflux hydronephrosis; previous UTI more than once; haematuria more than once; active UTI; suspected prostate or bladder cancer, AUR unrelated to prostate pathology; neurogenic bladder; urethral stricture; coagulates; bladder lithiasis; anaesthetic procedures from major surgery; patient unable to informed consent; postural hypotension; previous syncope; severe heart failure or instability; cholinergics; anticholinergics; MAO inhibitors; severe liver failure | Age 65 years |
| ***Sharifi 2014*** *[37]* | First episode AUR due to BPH (< 65 years of age) | Urethral stricture; previous urological surgery; chronic renal failure; diabetes; bladder or prostate cancer; prolonged constipation; active UTI; gross haematuria; nitrate consumption; addiction to opium or alcohol; RV > 1000 mL; previous use of alpha-blocker or 5-alpha-reductase inhibitor | Age 60 years<br>Prostate size 54 mL<br>RV 728 mL |
| ***Zhengyong 2014*** *[38]* | First episode AUR due to BPH | UTI; urological tumours; prostatic malignancy; urethral stricture; chronic urinary retention; urolithiasis; drug abuse; diabetes; neurogenic lower urinary tract dysfunction; previous prostatic surgery; recent urological manipulation | Age 66 (median)<br>IPSS 19<br>Prostate size 52 g<br>RV 852 mL<br>LUTS duration 15 months<br>IPP 10 mm<br>PSA 7.1 ng/mL |
| ***Elbendary 2013*** *[39]* | First episode AUR due to BPH | Neurological disorder; albumin < 30 g/L; prothrombin activity < 75%; creatinine > 41.4 mg/dL; suspected prostate cancer; concomitant bladder or urethral disease | Age 64 years<br>Previous alpha-blocker 16.0%<br>Previous alpha-blocker + finasteride 30.2%<br>Diabetes 81.1%<br>RV 763 mL<br>Prostate size 62 g |

| | | | |
|---|---|---|---|
| ***Kumar 2013*** *[40]* | First episode AUR (> 50 years of age), RV 400–1000 mL | Suprapubic catheterization; post-operative retention; clot retention; unable to consent; creatinine > 1.4 mg/dL; liver disease; neurological disease; urethral stricture; previous prostatic or bladder neck surgery; prostate carcinoma; unstable angina; recent myocardial infarction; cerebrovascular accidents with residual disesase; transient ischaemic attacks last 6 months; orthostatic hypotension; allergy to silodosin; current use of ketoconazole, chlarithromycin, itraconazole, nefazodone, ritonavir, or alpha-blocker | Age 65 years<br>LUTS duration 6 months<br>RV 770 mL<br>IPSS 26<br>Creatinine 1.1 mg/dL<br>PSA 3.7 ng/mL<br>Prostate size 43 mL<br>IPP > 10 mm 51.7%<br>Alcohol intake 11.7%<br>Constipation 36.7%<br>Diabetes 11.7% |
| ***Lodh 2013*** *[68]* | First AUR secondary to BPH (age ≥ 45 years) | Medical treatment for BPH and overactive bladder, gross hematuria/UTI/chronic constipation, suspected carcinoma of the prostate, urethral stricture urethra/vesical calculus, failed catheterization, bed-ridden/severely comorbid patients, recent anesthesia/surgery, neurological disorders affecting continence | Age 68.0 years<br>IPSS 22.3<br>Prostate size 53.4 mL<br>RV 588 mL<br>Transition zone volume 22.2 mL<br>Transition zone index 0.39<br>PSA 5.8 ng/mL |
| ***Mahadik 2013*** *[69]* | AUR due to BPH | Diagnosis other than BPH; RV > 1200 mL, neurological history; recent use of 5-alpha-reductase inhibitors, alpha-blockers, tricyclic antidepressants, anticholinergics, sympatomimetics, or first-generation antihistamines | Age 65.9 years<br>Prostate size 45 mL (median)<br>RV 855 mL<br>PSA 7.4 ng/mL<br>LUTS duration 2.1 months |
| ***Sharis 2013*** *[82]* | First episode AUR | Prostate cancer; recurrent or chronic urinary retention; UTI; renal impairment; bladder or urethral stone; bilateral hydronephrosis; neurological disorders affecting continence | Age 70 years<br>Prostate size 17 g<br>IPP > 10 mm 53.1% |

| | | | |
|---|---|---|---|
| ***Fitzpatrick 2012*** *[12]*<br><br>*Emberton 2008 [70]*<br><br>*Desgrandchamps 2006 [71]* | Painful AUR due to BPH | - | Age 70 years (median)<br>Severe LUTS 29.2%<br>Previous AUR 16.5%<br>Prostate size > 50 g 43.1% |
| ***Park 2012*** *[91]* | First episode AUR and could be followed-up for 4 weeks | Previous surgery of lower urinary tract; AUR secondary to specific cause such as urethral stricture or stone | 73 years<br>Prostate size 48 mL<br>RV 680 mL<br>Previous BPH 53.5%<br>Alcohol intake 8.0%<br>UTI 4.0%<br>PSA 5.8 ng/mL |
| ***Bhomi 2011*** *[72]* | First episode AUR due to BPH (≥ 50 years of age) | Absolute indication for prostatectomy; neurological impairment; suprapubic catheterization; gross haematuria; UTI; anticholinergics; severe co-morbidities | Age 67 years<br>LUTS duration 9 months<br>IPSS 17<br>RV 841 mL<br>Prostate size 59 g<br>IPP 9 mm |
| ***Zeif 2010*** *[83]* | AUR | Previous prostate cancer; clot retention; catheter-related problems | Age 71 years<br>RV 832 mL<br>UTI 13.0%<br>PSA 96.3 ng/mL<br>CRP 48.3 mmol/L<br>Prostate cancer 9.0% |
| ***Agrawal 2009*** *[41]* | First episode AUR due to BPH (48–90 years of age) | Initial RV < 500 mL or > 1500 mL; previous prostatic surgery; malignancy; other bladder disease; retention due to medication; creatinine > 2 mg/dL; macroscopic haematuria; UTI; hepatic impairment | Age 70.7 years<br>RV 462 mL |

| | | | |
|---|---|---|---|
| ***Daly 2009*** *[73]* | AUR | Other urologic disease, including previous AUR; prostatic malignancy; previous lower urinary tract surgery | Age 72 years<br>RV 936 mL<br>PSA 2.9 ng/mL |
| ***Rasner 2009*** *[88]* | AUR due to BPH | - | Age 71 years<br>Prostate size 59 mL<br>RV 802 mL |
| ***Tiong 2009*** *[42]* | First painful AUR due to BPH (> 55 years of age), RV 500–1000 mL | Unable to consent; creatinine > 120 mmol/L; hepatic disease; significant neurological disease; urethral stricture; previous prostatic or bladder neck surgery; prostate carcinoma; unstable angina; myocardial infarction; cerebrovascular accident with residual neurology; transient ischaemic attack last 6 months; orthostatic hypotension; hypersensitivity to alpha-blockers; MAO-inhibitors; cholinergics; anticholinergics; other alpha-blocking drugs; suprapubic catheterization; postoperative retention; clot retention | Age 72 years<br>Diabetes 20.3%<br>Cerebrovascular accident 14.1%<br>Constipation 12.5%<br>IPSS 17<br>Prostate size 2.9 finger breadths<br>IPP > 10 mm 32.8%<br>RV 839 mL<br>PSA 28.9 ng/mL<br>Creatinine 101 mmol/L |
| ***Panda 2008*** *[84]* | AUR due to BPH | - | Age 67 years |
| ***Pandit 2008*** *[74]* | AUR due to BPH | Non-prostate related surgery; anaesthesia; sympathomimetics; anticholinergics; antihistamines; neurogenic AUR; UTI | Age 66.8 years<br>IPSS 15.9<br>Prostate size 53.1 mL |
| ***Tsui 2008*** *[89]* | First episode AUR due to BPH treated with catheter and terazosin 2 or 4 mg | AUR from other causes than BPH; already on alpha-blocker; previous AUR; patients not attending follow-up appointment | Age 72.3 years |
| ***Al-Hashimi 2007*** *[43]* | First episode spontaneous AUR due to BPH, RV < 1500 mL | Precipitated AUR or AUR related to other than BPH: postoperative, drug precipitated, neurogenic causes, prostatic cancer, vesical stones, urethral stricture, lower urinary tract surgery, etc | Age 69 years<br>Prostate size 65 mL<br>UTI 8.4%<br>PSA 2.2 ng/dL |

| | | | |
|---|---|---|---|
| ***Mariappan 2007*** *[75]* | AUR due to BPH (≥ 50 years of age), RV < 1500 mL | Abnormal renal function; previously failed TWOC; prostatic or urethral surgery (including prostate biopsy); urethral stricture; suprapubic catheterization; neurological disease; severe comorbidities; prostate cancer; anticholinergics | Age 70 years<br>Prostate size 68 mL<br>RV 834 mL<br>IPP 12 mm |
| ***Gopi 2006*** *[76]* | AUR | Chronic retention; macroscopic haematuria; sepsis; UTI; abnormal creatinine | Age 69 years (median)<br>RV 900 mL (median) |
| ***Park 2006*** *[90]* | AUR due to BPH | Neuropathic bladder; anticholinergics; previous prostate or bladder surgery | Age 71 years<br>IPSS 21<br>Prostate size 42 mL<br>RV 748 mL<br>PSA 5.3 ng/mL<br>UTI 11.9% |
| ***Lucas 2005*** *[44]*<br><br>*Lucas 2002 [45]* | AUR due to BPH (51–91 years of age) | RV < 500 mL or > 1500 mL; renal or hepatic dysfunction; previous urinary tract surgery; other bladder diseases; malignancy; retention-enhancing medication; allergies; severe cardiac disease | Age 69 years |
| ***Lorente Garín 2004*** *[50]* | First AUR due to BPH | | Age 73 years<br>IPSS 17<br>Prostate size 53 mL<br>RV 966 mL |

| | | | |
|---|---|---|---|
| ***McNeill 2004*** *[46]*<br><br>*McNeill 2005 [47]*<br><br>*Hargreave 2003 [48]*<br><br>*McNeill 2003 [49]* | AUR due to BPH (≥ 51 years of age), RV 500–1500 mL | Unability to consent; mental disorders; unreliability to understand trial requirements and comply with treatment regimen; participation in clinical trial the previous 3 months; neurogenic bladder dysfunction; isolated bladder neck disease; acute or chronic prostatitis; diagnosed or suspected carcinoma of prostate; history of prostatic and urethral surgery; urethral stricture; bladder stones; clot retention; AUR not related to BPH (postoperative retention following major abdominal/pelvic surgery, anesthesia) within the 14 last days; Parkinson's disease; insulin dependent diabetes; multiple sclerosis; stroke or myocardial infarction within the previous 6 months; hepatic abnormalities; neutropenia; renal insufficiency; unstable or severe heart failure; postural hypotension or syncope; hypersensitivity to alpha-blockers; evolutive neoplastic disease; sympathomimetics within the previous week; 5-alpha-reductase inhibitors within the previous 3 months; alpha-1-blockers in the previous month; tricyclic antidepressants, anticholinergics, sympathomimetics or first generation antihistamines within the previous month; disopyramide. | Age 69 years<br>RV 978 mL<br>LUTS duration 33 months<br>Constipation 11.4%<br>UTI 2.8% |
| ***Hua 2003*** *[51]* | First AUR due to BPH | Previous prostate surgery; having taken alpha-blockers | Age 71 years<br>RV 860 mL<br>Prostate size 52 mL |
| ***Tan 2003*** *[85]* | First AUR episode (> 50 years of age) | Prostatic cancer; recurrent or chronic urinary retention; UTI; bilateral hydronephrosis; renal impairment; neurological disease such as cerebrovascular accidents or parkinsonism | Age 70 years<br>Prostate size 39 g<br>RV 829 mL<br>PSA 15 ug/L<br>IPP > 10 mm 45.0% |
| ***Shah 2002*** *[52]* | AUR | Cardiac disease contra-indicating use of alpha-blockers; medical therapy for bladder outflow obstruction; bladder calculi; prostate cancer; renal impairment; urethral stricture; UTI; neurogenic bladder dysfuncion; bladder tumour; clot retention; unability to informed consent | Age 68.7 years<br>RV 977 mL |
| ***Abeygunasekera 2001*** *[77]* | First AUR having a clinically enlarged prostate | | Age 68 years<br>Prostate size > 35 g 6.4%<br>LUTS > 4 weeks 87.2% |

| | | | |
|---|---|---|---|
| ***Bowden 2001*** *[53]* | AUR due to BPH | | Age 71.1 years<br>RV 854 mL |
| ***Kim 2001*** *[78]* | AUR | Creatinine >2.0; prostate cancer; suspected malignancy on digital rectal examination; neurogenic voiding dysfunction; anticholinergic medication; suprapubic catheter | Age 71 years<br>RV 790 mL<br>Prior treatment for BPH 27.3%<br>IPSS 17 |
| ***Perepanova 2001*** *[54]* | AUR due to BPH | Bladder stones; macrohaeamaturia; urethral stricture; bladder tumours; prostate cancer; infectious-inflammatory processes in the urinary organs; prostate surgery | Age 68.4 years<br>Prostate size 71 mL<br>LUTS duration 5.0 years |
| ***Kumar 2000*** *[86]* | Primary AUR | Lower urinary tract pathology that might have influenced the natural course of BPH, e.g., prostate carcinoma; high age-specific PSA; clot retention; urethral stricture; pelvic colon malignancy; shingles; carcinoma of penis; meatal stenosis; phimosis; neuropathic bladder | Age 74 years<br>RV 926 mL<br>Prostate size 21 g<br>LUTS > 6 months 30.0%<br>Previous AUR 7.5%<br>UTI 12.5%<br>Constipation 40.0%<br>Alcohol intake 2.5%<br>Anticholinergic medication 7.5%<br>On alpha-blocker 20.0% |
| ***Lim 1999*** *[59]* | AUR | <50 years of age; RV <200 mL; creatinine >200 mmol/L; clot retention; prostatic carcinoma; bladder stone; bilateral hydronephrosis | Age 70 years<br>PSA 21.9 ug/L<br>RV 755 mL<br>IPSS > 20 16.2% |

| | | | |
|---|---|---|---|
| ***McNeill 1999*** *[55]*<br>*McNeill 2004 [56]*<br>*McNeill 1998 [57]*<br>*McNeill 2000 [58]* | AUR due to BPH (≥ 55 years of age), RV 500–1500 mL | Unability to give informed consent; significant renal and/or hepatic disease; depressive illness on medication; extra-pyramidal disorders; neurological disease, e.g. MS, spinal injury; urethral stricture; dipstick detected UTI, acute or chronic prostatitis;<br>prostatic or bladder neck surgery; carcinoma of the prostate; unstable angina pectoris; myocardial infarction; transient ischaemic attacks; cerebrovascular accident or congestive cardiac failure the previous 6 months; orthostatic hypotension; MAO inhibitors; cholinergic or anticholinergic drugs; calcium-channel blockers, alpha-blocking drugs; hypersensitivity to alfuzosin or alpha-blockers; suprapubic catheterization; postoperative retention after major abdominal/pelvic surgery; clot retention | Age 70 years<br>Prostate size > 51 g 28.4%<br>RV 981 mL |
| ***Hastie 1990*** *[87]* | AUR | Postoperative retention; chronic retention; clot retention; urethral stricture; neuropathic bladder | Age 72 years<br>RV 909 mL<br>Prostate size 33 g<br>LUTS 60.5%<br>UTI 19.7%<br>Gross constipation 7.9% |

AUA: American Urological Association; AUR: acute urinary retention; BWT: bladder wall thickness; BPH: benign prostate hyperplasia; CRP: C-reactive protein; DRE: digital rectal examination; DWT: detrusor wall thickness; IPP: intravesical prostatic protrusion; IPSS: International Prostate Symptom Score; LUTS: lower urinary tract symptoms; MAO: monoamine oxidase; PSA: prostate specific antigen; RCT: randomized controlled trial; RV: residual volume; TWOC: trial without catheter; UTI: urinary tract infection.
For continuous variables, means are stated unless specified otherwise.
