# Supplementary material for: Immediate or delayed trial without catheter in acute urinary retention in males: A systematic review
Source: BJUI Compass. 2024 May 14;5(8):732–47. doi: 10.1002/bco2.369 (PMC11327489; doi:10.1002/bco2.369)
Supplement: Supplementary file 4 — Table S4. Studies reporting secondary outcomes – complications and adverse events. [file BCO2-5-732-s012.pdf]

Supplementary table 4. Studies reporting secondary outcomes – complications and adverse events

Study	Intervention	Complications/adverse events
***Salem Mohamed 2018** [33]*	TWOC day 3 + tamsulosin 0.4 mg + levofloxacin 500 mg<br>TWOC day 7 + tamsulosin 0.4 mg + levofloxacin 500 mg	TWOC day 3 (n = 30) vs TWOC day 7 (n = 30): any catheter complication 16.7% vs 43.3% (p = 0.02); catheter obstruction 3.3% vs 10% (p = 0.3); UTI 6.7% vs 33.3% (p = 0.01); haematuria 3.3% vs 16.7% (p = 0.08); catheter leakage 3.3% in both groups.
***Zhengyong 2014** [38]*	TWOC day 7 + tamsulosin 0.2 mg + finasteride 5 mg + bladder training<br>TWOC day 7 + tamsulosin 0.2 mg + finasteride 5 mg + free drainage	Bladder training group (n = 440) vs free drainage group (n = 405):<br>UTI 6.8% vs 6.4% (p = 0.82); urine leakage 7.5% vs 6.9% (p = 0.74); catheter obstruction 1.8% vs 1.5% (p = 0.70); haematuria 1.1% vs 0.7% (p = 0.55)
***Elbendary 2013** [39]*	TWOC day 7 + tamsulosin 0.4 mg + ketoconazole 200 mg<br>TWOC day 7 + tamsulosin 0.4 mg + placebo	No de novo erectile dysfunction after catheter removal
***Fitzpatrick 2012** [12]*	TWOC day 5 (median) + alpha-blocker	TWOC ≤ 3 days (n = 1853) vs TWOC > 3 days (n = 2638):<br>any adverse event 19.7% vs 33.8% (p < 0.001); haematuria 9.6% vs 10.4% (p = 0.35); asymptomatic bacteriruria 5.7% vs 13.5% (p < 0.001); lower UTI 3.4% vs 7.2% (p < 0.001); urosepsis 0.6% vs 1.2% (p = 0.06); urine leak 3.7% vs 6.9% (p < 0.001); catheter obstruction 0.8% vs 3.1% (p < 0.001); other adverse events 1.0% vs 1.7% (p = 0.05); prolongation of hospitalization for adverse event 3.5% vs 5.2% (p = 0.007)
***Al-Hashimi 2007** [43]*	TWOC day 3 + alfuzosin 10 mg<br>TWOC day 3 + placebo	Positive urine cultures at presentation in 19/224 (8.4%) and at TWOC in 13/107 (12.1%) (p > 0.05)
***McNeill 2004** [46]*	TWOC day 2 + alfuzosin 10 mg<br>TWOC day 2 + placebo	2/363 patients (0.6%) withdrew due to catheter related infection, one in each study arm

TWOC: trial without catheter; UTI: urinary tract infection.
