# Supplementary material for: Immediate or delayed trial without catheter in acute urinary retention in males: A systematic review
Source: BJUI Compass. 2024 May 14;5(8):732–47. doi: 10.1002/bco2.369 (PMC11327489; doi:10.1002/bco2.369)
Supplement: Supplementary file 5 — Table S5. Studies reporting secondary outcomes – patient satisfaction. [file BCO2-5-732-s009.pdf]

Supplementary table 5. Studies reporting secondary outcomes – patient satisfaction

Study	Intervention	Patient satisfaction
Hagiwara 2016 *[66]*	TWOC day 14 + silodosin 4 mg x2 + dutasteride 0.5 mg	Median IPSS QoL at presentation 5, 2 weeks later 3 ($p < 0.001$), 12 weeks after presentation 1 ($p < 0.001$)
Lorente Garín 2004 *[50]*	TWOC day 7 + doxazosin 4 mg<br>TWOC day 7	Mean IPSS QoL at presentation 4.0, at catheter removal 3.9
Kim 2001 *[78]*	TWOC day 7 + tamsulosin 0.4 mg	Mean IPSS QoL at initial evaluation 3.9 (SD 1.9); two weeks after successful TWOC 2.7 (SD 1.8)
Kumar 2000 *[86]*	TWOC day 2	Mean IPSS QoL 4 months after successful TWOC 1.0

IPSS QoL: International Prostate Symptom Score – Quality of Life Index; SD: standard deviation; TWOC: trial without catheter.
