# Supplementary material for: Immediate or delayed trial without catheter in acute urinary retention in males: A systematic review
Source: BJUI Compass. 2024 May 14;5(8):732–47. doi: 10.1002/bco2.369 (PMC11327489; doi:10.1002/bco2.369)
Supplement: Supplementary file 6 — Figure S1. Risk of bias assessment in the included randomized controlled trials, performed in RoB 2.0. 21 [file BCO2-5-732-s011.pdf]

Study ID	D1	D2	D3	D4	D5	Overall
Djavan 1998	!	+	+	+	+	!
Taube 1989	!	+	+	+	+	!

+ Low risk
! Some concerns
- High risk

D1	Randomisation process
D2	Deviations from the intended interventions
D3	Missing outcome data
D4	Measurement of the outcome
D5	Selection of the reported result
