# Supplementary material for: Immediate or delayed trial without catheter in acute urinary retention in males: A systematic review
Source: BJUI Compass. 2024 May 14;5(8):732–47. doi: 10.1002/bco2.369 (PMC11327489; doi:10.1002/bco2.369)
Supplement: Supplementary file 8 — Figure S3. Risk of bias assessment in the included cohort studies reporting only immediate TWOC, performed in ROBINS‐I. 22 [file BCO2-5-732-s002.pdf]

Study ID	D1	D2	D3	D4	D5	D6	D7	Overall
Li 2009	!!	+	+	+	+	+	+	!!
Chan 1996	!!	+	+	!!	+	+	+	!!
Klarskov 1987	!!	!!	!!	+	+	+	+	!!
Breum 1982	!!	!!	!	+	!	+	+	!!

+	Low risk
!	Moderate risk
!!	Serious risk
-	Critical risk

D1	Bias due to confounding
D2	Bias in selection of participants into the study
D3	Bias in classification of interventions
D4	Bias due to deviations from intended interventions
D5	Bias due to missing data
D6	Bias in measurement of outcomes
D7	Bias in selection of the reported results
