# Supplementary material for: Immediate or delayed trial without catheter in acute urinary retention in males: A systematic review
Source: BJUI Compass. 2024 May 14;5(8):732–47. doi: 10.1002/bco2.369 (PMC11327489; doi:10.1002/bco2.369)
Supplement: Supplementary file 9 — Figure S4. Risk of bias assessment in the included cohort studies reporting only delayed TWOC, performed in ROBINS‐I. 22 [file BCO2-5-732-s008.pdf]

Study ID	D1	D2	D3	D4	D5	D6	D7	Overall
Khadka 2021	!!	+	+	!!	+	+	+	!!
Phuong Hoai 2021	!!	+	+	!!	+	+	+	!!
Jha 2020	!!	+	+	!!	+	+	+	!!
Gas 2019	!!	!!	!!	!!	!	+	+	!!
Kurniasari 2019	!!	+	+	+	+	+	+	!!
Vella 2019	!!	+	+	!!	+	+	+	!!
Das 2018	!!	+	+	!!	+	+	+	!!
Salem Mohamed 2018	!!	+	+	!!	+	+	+	!!
Bansal 2017	!!	+	+	!!	+	+	+	!!
Farelo-Trejos 2017	!!	+	+	!!	+	+	+	!!
Ferdian 2016	!!	+	+	!!	+	+	+	!!
Hagiwara 2016	!!	+	+	!!	+	+	+	!!
Tang 2015	!!	!!	!!	!!	+	+	+	!!
Green 2014	!!	!!	!!	!!	+	+	+	!!
Kara 2014	!!	+	+	!!	+	+	+	!!
Maldonado-Ávila 2014	!!	+	+	!!	+	+	+	!!
Sharifi 2014	!!	+	+	!!	+	+	+	!!
Zhengyong 2014	!!	+	+	!!	+	+	+	!!
Elbendary 2013	!!	+	+	!!	+	+	+	!!
Kumar 2013	!!	+	+	!!	+	+	+	!!
Lodh 2013	!!	+	+	!!	+	+	+	!!
Mahadik 2013	!!	+	+	!!	!!	+	+	!!
Sharis 2013	!!	+	+	+	+	+	+	!!
Fitzpatrick 2012	!!	+	+	!!	+	+	+	!!
Park 2012	!!	!!	+	+	+	+	+	!!
Bhomi 2011	!!	+	+	!!	+	+	+	!!
Zeif 2010	!!	+	+	+	+	+	+	!!
Agrawal 2009	!!	+	+	!!	+	+	+	!!
Daly 2009	!!	+	+	!!	+	+	+	!!
Rasner 2009	!!	!!	+	!!	+	+	+	!!
Tiong 2009	!!	+	+	!!	+	+	+	!!
Panda 2008	!!	+	+	+	+	+	+	!!
Pandit 2008	!!	+	+	!!	!!	+	+	!!
Tsui 2008	!!	!!	+	!!	!!	+	+	!!
Al-Hashimi 2007	!!	+	+	!!	+	+	+	!!
Mariappan 2007	!!	+	+	!!	+	+	+	!!
Gopi 2006	!!	+	+	!!	+	+	+	!!
Park 2006	!!	!!	+	!!	+	+	+	!!
Lucas 2005	!!	+	+	!!	!	+	+	!!
McNeill 2004	!!	+	+	!!	+	+	+	!!
Lorente Garin 2004	!!	+	+	!!	+	+	+	!!
Hua 2003	!!	+	+	!!	+	+	+	!!
Tan 2003	!!	+	+	+	+	+	+	!!
Shah 2002	!!	+	+	!!	!!	+	+	!!
Abeygunasekera 2001	!!	+	+	!!	!	+	+	!!
Bowden 2001	!!	+	+	!!	+	+	+	!!
Kim 2001	!!	+	+	!!	+	+	+	!!
Perepanova 2001	!!	+	+	!!	+	+	+	!!
Kumar 2000	!!	+	+	!	+	+	+	!!
Lim 1999	!!	+	+	+	+	+	+	!!
McNeill 1999	!!	+	+	!!	+	+	+	!!
Hastie 1990	!!	+	!	+	+	+	+	!!

- + Low risk
- ! Moderate risk
- !! Serious risk
- - Critical risk

D1	Bias due to confounding
D2	Bias in selection of participants into the study
D3	Bias in classification of interventions
D4	Bias due to deviations from intended interventions
D5	Bias due to missing data
D6	Bias in measurement of outcomes
D7	Bias in selection of the reported results
