# Supplementary figures and images for: Immediate or delayed trial without catheter in acute urinary retention in males: A systematic review
Source: BJUI Compass. 2024 May 14;5(8):732–47. doi: 10.1002/bco2.369 (PMC11327489; doi:10.1002/bco2.369)

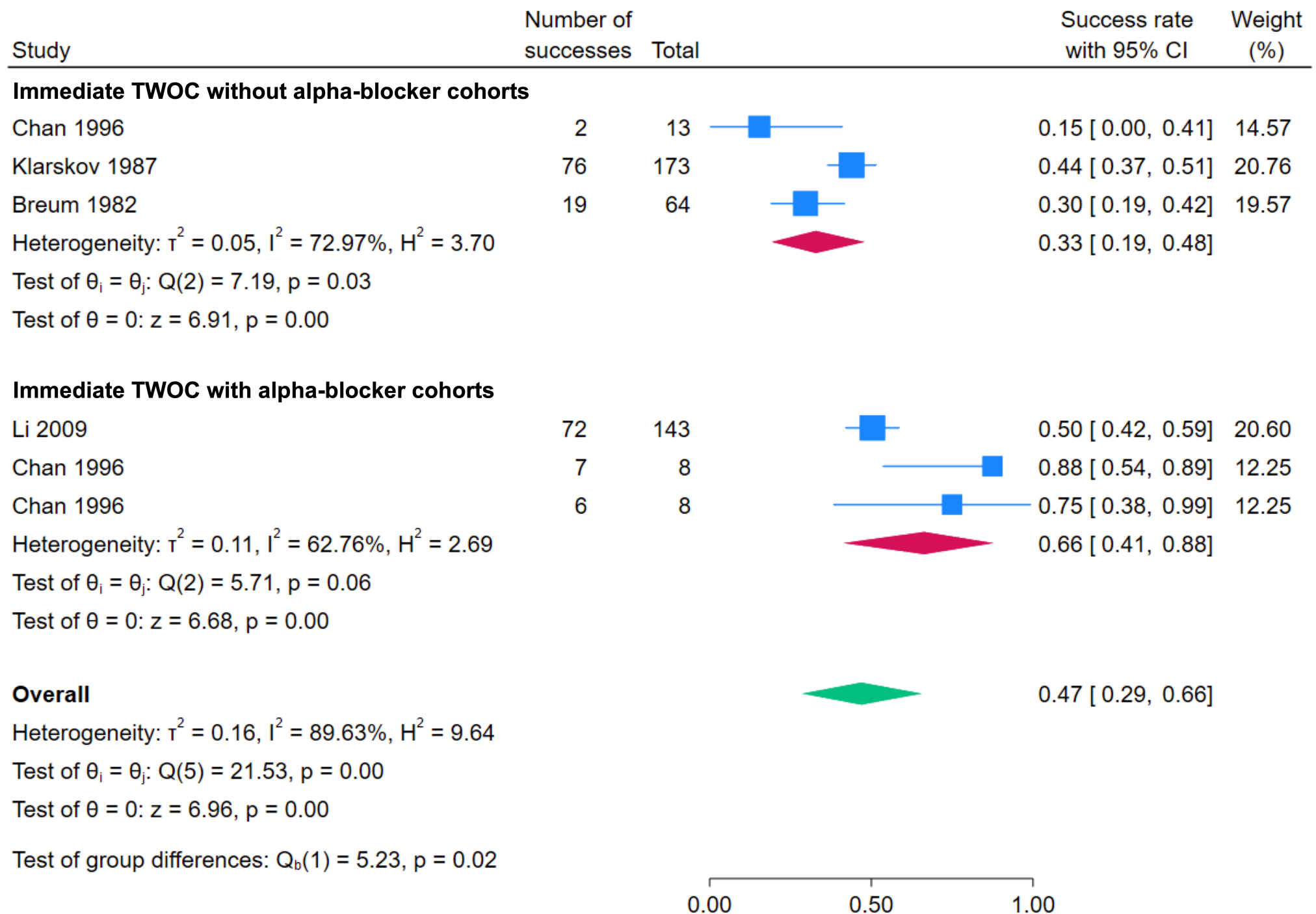

Supplement: Supplementary file 10 — Figure S5. Meta‐analysis of cohort studies reporting success rates for immediate TWOC. [file BCO2-5-732-s005.pdf]

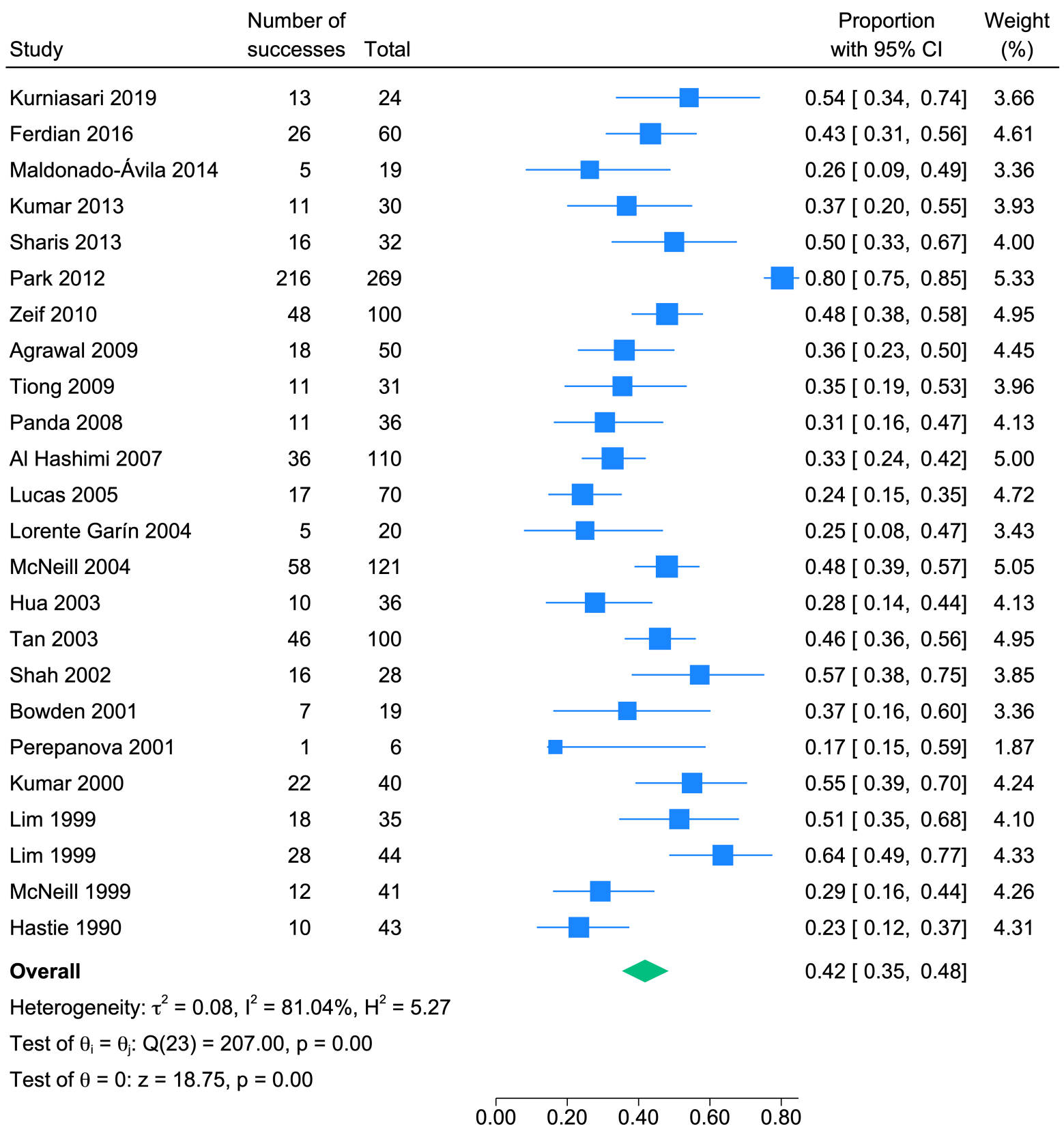

Random-effects REML model

Supplement: Supplementary file 11 — Figure S6. Meta‐analysis of cohort studies reporting success rates for delayed TWOC for patients not given alpha‐blockers. [file BCO2-5-732-s006.pdf]

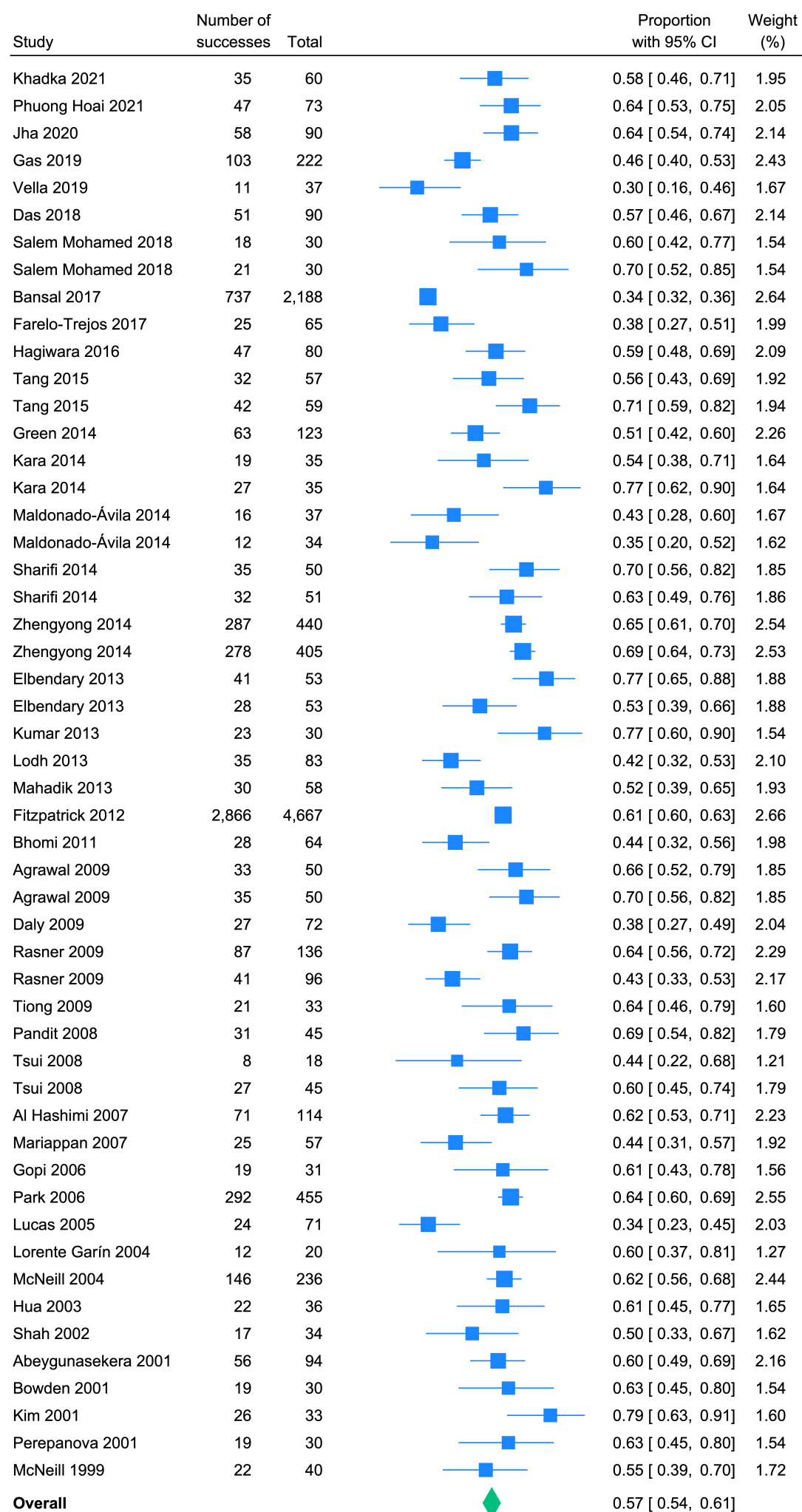

Heterogeneity:  $\tau^2 = 0.04$ ,  $I^2 = 88.34\%$ ,  $H^2 = 8.58$

Test of  $\theta_1 = \theta_0$ :  $Q(51) = 708.93$ ,  $p = 0.00$

Test of  $\theta = 0$ :  $z = 45.67$ ,  $p = 0.00$

0.20 0.40 0.60 0.80 1.00

Supplement: Supplementary file 12 — Figure S7. Meta‐analysis of cohort studies reporting success rates for delayed TWOC for patients given alpha‐blockers. [file BCO2-5-732-s010.pdf]
